# Draft Crystal Structure of the Vault Shell at 9-Å Resolution

**DOI:** 10.1371/journal.pbio.0050318

**Published:** 2007-11-27

**Authors:** Daniel H Anderson, Valerie A Kickhoefer, Stuart A Sievers, Leonard H Rome, David Eisenberg

**Affiliations:** 1 Howard Hughes Medical Institute, University of California Los Angeles, Los Angeles, California, United States of America; 2 Department of Biological Chemistry, David Geffen School of Medicine, University of California Los Angeles, Los Angeles, California, United States of America; 3 California NanoSystems Institute, University of California Los Angeles, Los Angeles, California, United States of America; 4 Department of Chemistry and Biochemistry, University of California Los Angeles, Los Angeles, California, United States of America; Brandeis University, United States of America

## Abstract

Vaults are the largest known cytoplasmic ribonucleoprotein structures and may function in innate immunity. The vault shell self-assembles from 96 copies of major vault protein and encapsulates two other proteins and a small RNA. We crystallized rat liver vaults and several recombinant vaults, all among the largest non-icosahedral particles to have been crystallized. The best crystals thus far were formed from empty vaults built from a cysteine-tag construct of major vault protein (termed cpMVP vaults), diffracting to about 9-Å resolution. The asymmetric unit contains a half vault of molecular mass 4.65 MDa. X-ray phasing was initiated by molecular replacement, using density from cryo-electron microscopy (cryo-EM). Phases were improved by density modification, including concentric 24- and 48-fold rotational symmetry averaging. From this, the continuous cryo-EM electron density separated into domain-like blocks. A draft atomic model of cpMVP was fit to this improved density from 15 domain models. Three domains were adapted from a nuclear magnetic resonance substructure. Nine domain models originated in ab initio tertiary structure prediction. Three C-terminal domains were built by fitting poly-alanine to the electron density. Locations of loops in this model provide sites to test vault functions and to exploit vaults as nanocapsules.

## Author Summary

Vaults are large barrel-shaped particles found in the cytoplasm in all mammalian cells, which may function in innate immunity. As naturally occurring nanoscale capsules, vaults may be useful objects to engineer as delivery vehicles. In this study, we propose an atomic structure for the thin outer shell of the vault. Using x-ray diffraction and computer modeling, we have inferred a draft atomic model for the major vault protein, which forms the shell-like enclosure of the vault. The shell is made up of 96 identical protein chains, each of 873 amino acid residues, folded into 14 domains. Each chain forms an elongated stave of half the vault, as well as the cap of the barrel-like shell. Our draft atomic model is essentially an atomic-level model for the entire 9.3-MDa vault shell, which offers a guide for protein engineering to test vault functions and to exploit vault particles as nanocapsules.

## Abstract 3

A draft atomic structure has been determined for the 9.3-MDa protein shell of the vault cytoplasmic particle, revealing stave-like polypeptides forming the barrel-like structure of the vault.

## Introduction

Vault ribonucleoprotein particles are found in the cytoplasm of most eukaryotic cells [1]. Ninety-six copies of major vault protein (MVP; 95.8 kDa) form the thin, hollow vault shell with dimensions reported as 725 × 410 × 410 Å^3^ [2]. The MVP shell encapsulates a 50 × 10^6^–Å^3^ interior volume that contains 2–4 copies of telomerase associated protein 1 (TEP1; 290 kDa), about 12 copies of an enzyme, poly(ADP-ribose)polymerase (VPARP; 193 kDa), and 8–16 copies of a small untranslated RNA. The mass of a rat liver vault is about 13 × 10^6^ Da [3]. Most eukaryotic cells contain upwards of 10,000 copies of vaults [4]. MVP expressed in insect cells self-assembles into vault shells [5].

Vaults were recently shown to have a protective role in innate immunity [6]. MVP co-localized with Pseudomonas aeruginosa in lung epithelial cells at an early stage of infection, and MVP knockout mice [7], which do not form vault particles, were shown to be more susceptible to bacterial lung infection. Vaults had previously been implicated in multidrug resistance [8] and cellular signaling [9–12]; however, their exact role in any of these pathways remains elusive.

**Figure 1:**
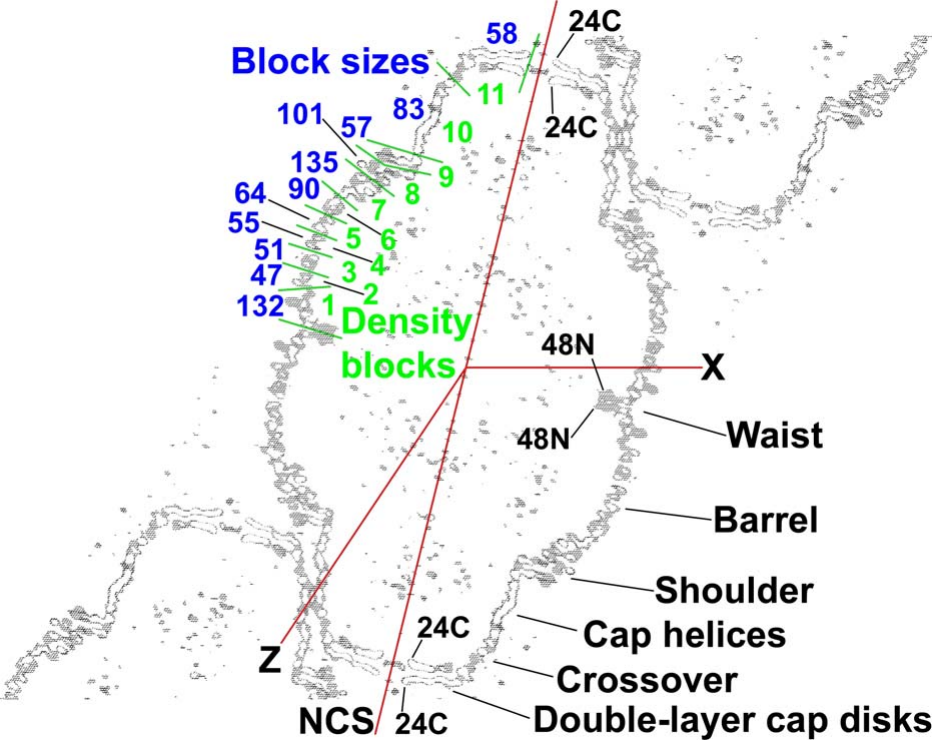
Thin Section of Crystalline Vault Electron Density The red lines show the crystal *x* and *z* directions, and the direction of the high-symmetry vault axis (marked NCS for noncrystallographic symmetry). The two neighboring vaults at upper right and lower left are related to the central vault by translations along the crystal *z* direction. The vault and the map are centered at (0,0,0) (contoured box is 530 Å along the crystal *x-*axis, 5-Å thick on *y*, and 845 Å along *z*). Regions of the vault discussed in the text are labeled at lower right. The vault model is 675 Å tip-to-tip and 417 Å in diameter at the widest part of the barrel. The 96 N termini are inside the vault at the waist region (marked 48N). Pairs of MVP chains become nonequivalent in the crossover zone as they approach the double-layer, C-terminal disk regions (C termini of the model are marked 24C). The vault model leaves ∼29-Å holes between C termini. The green lines at upper left mark the partitions between density blocks 1–11 used for “dot model refinement.” These partitions were chosen for convenience of handling files and do not match the cpMVP model domains (Table 1 and Figure 4). The blue numbers at upper left are density block size estimates: (873 residues) × (dots in block)/(total dots). The block size estimates were used for initial placement of cpMVP model domain 7. This figure, including the red and green lines, was made with XFIT of XtalView [40] and RENDER of Raster3D [44], and was labeled with Adobe Photoshop.

Vault structure has previously been probed by transmission electron microscopy, cryo-electron microscopy (cryo-EM), and nuclear magnetic resonance (NMR). Multi-image averaging greatly clarified the cryo-EM image of the MVP shell [1]. Vault anatomical terms, emerging from both earlier work and our own, are shown in Figure 1. Internal contents of rat vaults and new features of modified recombinant vaults have been localized by cryo-EM difference mapping. The RNA and a portion of TEP1 reside inside the vault near the ends of its two caps [13]. The N termini of MVP form the waist and extend toward the vault interior, and VPARP localizes onto the inner surfaces of the vault [2]. During our work, an MVP substructure was determined by NMR (residues 113–221 of human MVP [14]). Engineering of the vault by encapsulation of exogenous components has begun [15]; proteins can be targeted to the inside surface of the vault by expression as fusions with either the N terminus of MVP or a VPARP-derived targeting domain, and localization to the vault interior can be confirmed by cryo-EM difference mapping.

**Table 1:**
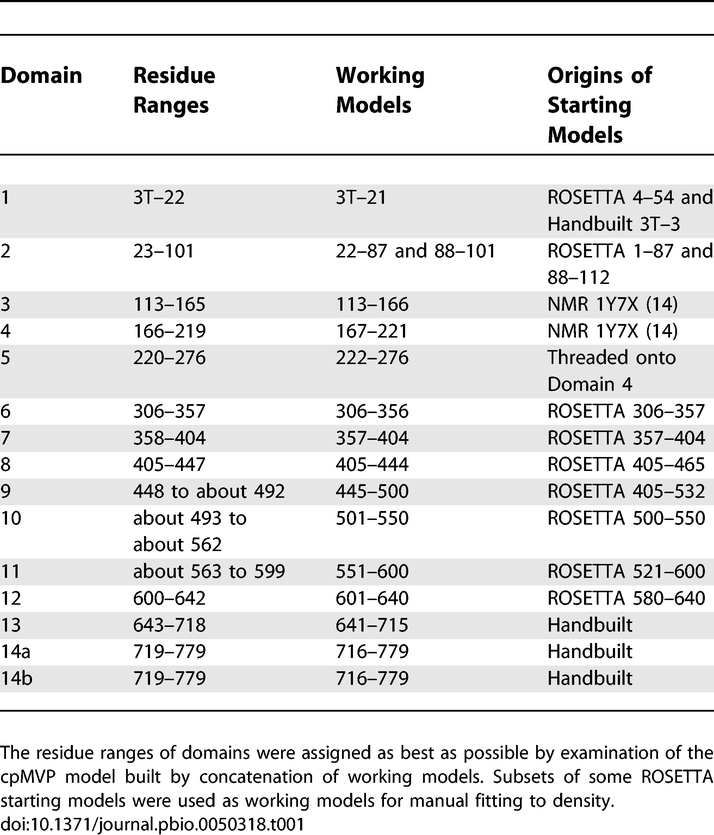
Domain Partitions

**Figure 2:**
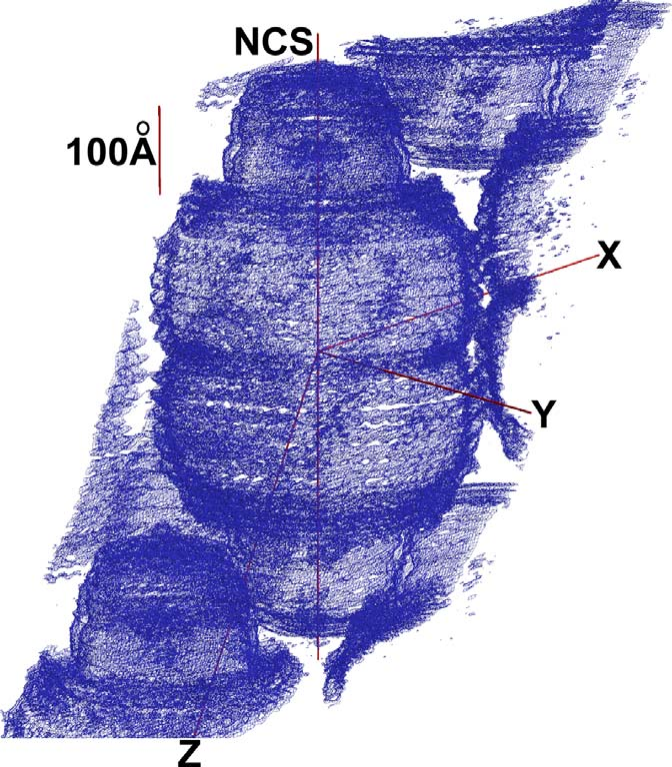
Overall View of the cpMVP Vault Averaged Electron Density Map, at about 9-Å Resolution, in the Context of the Crystal Packing This electron density map (wire frame representation) resulted from applying solvent flattening and a 48-fold rotational symmetry averaging to the featureless cryo-EM electron density. Separation into globules of density showed that the MVP chain folds into a series of domains. The short red line is a 100-Å scale bar. The line marked NCS shows the noncrystallographic symmetry axis used for phasing. One of the 48 2-fold axes through the vault waist is coincident with the crystal 2-fold in the *y* direction (perpendicular to NCS axis). The figure was made using XFIT of XtalView and RENDER of Raster3D, then labeled with Adobe Photoshop. A section through the top of this figure is part of Figure S1.

Extending the cryo-EM vault structure via crystallography to derive an atomic model is of great potential value in designing modifications of the vault structure and to elucidate function. The crystallographic difference-Fourier technique applied to future cocrystals could precisely localize internal vault components, while indicating their shapes and thus orientations relative to the MVP shell.

## Results

### Initial Phasing of x-Ray Reflections and Evidence of Domains

Phasing was initiated by manual placement of cryo-EM electron density of a half vault at a crystal 2-fold axis (see Methods). The phases, and thus the detail in the image of the vault, were initially improved by density modification using a single 48-fold rotational noncrystallographic symmetry (NCS) operator (marked NCS in Figure 1). NCS is symmetry of the vault that is not shared with the crystal. The results from testing parameters for averaging paralleled those reported for spherical viruses [16,17], except that the phases “condensed” into two pseudo-Babinet-inverse sets (see Methods and Figure S1). One phase set was selected because the map derived from it contained double-disk C-terminal structures that could plausibly contain 24-fold symmetric MVP chains in each layer (marked 24C in Figure 1).

The featureless cryo-EM electron density separated into globules in the more plausible 48-fold averaged electron density map (Figure 2), indicating more preferential cohesion within short segments of the MVP chain than between consecutive segments. This meant that the MVP monomer, at least below the C-terminal cap structure, folded into domains (see Figure 1 for initial partitions). The first averaging was not biased by prior expectation of domains. The density globules were spaced as would be backbone atoms with side chains between. The barrel portion of the vault appeared built from vertical “staves” of stacked domains. Observation of stacked domains parallels one conclusion of the NMR spectroscopists [14].

This initial 48-fold averaging was later improved by “dot model refinement,” applying concentric 24-fold (density block 11 in Figure 1) and 48-fold (density blocks 1–10 in Figure 1) NCS axes (see Methods) and domain-shaped “dot models” to re-initiate the phase sets. The enantiomer of the electron density map was assigned during model building.

### Construction of the Vault Model

**Figure 3:**
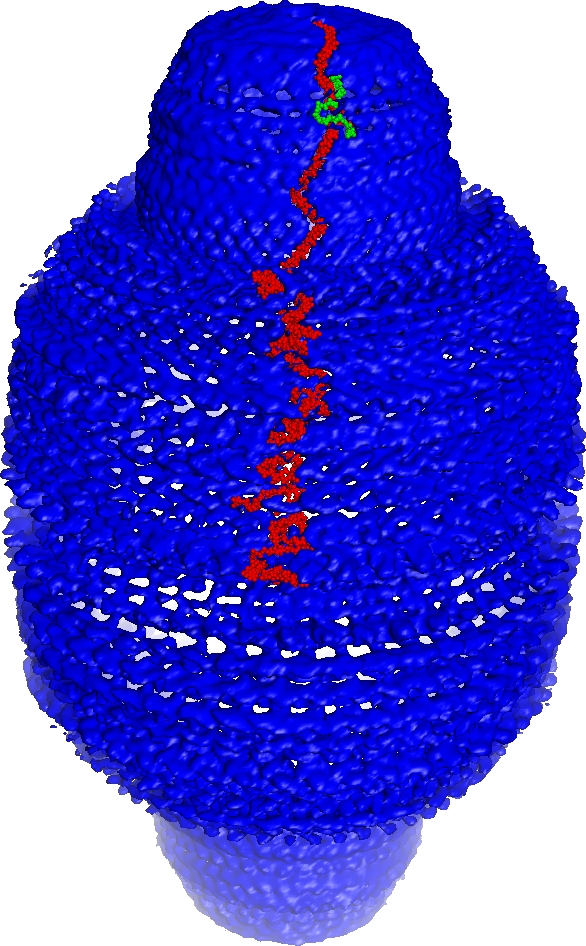
Overall View of Dot-Refined Vault Electron Density with the Unique Parts of the cpMVP Model Inserted One copy of the cpMVP model is shown as red atoms, from its N terminus at the waist to the crossover zone near the top (as in Figure 4). Two nonequivalent copies of cpMVP model are shown from the crossover to the C termini (the path of the green cpMVP model is mostly occluded; see Figure 1 for orientation). The electron density map coefficients were *F*_observed_, and the phase set was the enantiomer of the phases from the slow-averaged Dot Model 6. The contour level was 1.2σ. The electron density becomes less symmetric near crystal lattice contacts (left of center, foreground). The map and masks were produced with CCP4 programs [31]. Surrounding electron density was masked off to make this figure. The density around the cpMVP model was deleted with an inverse mask (inversion performed with MAMA [45]). The opaque iso-surface representation with “fog” representing distance was drawn with PyMOL [46].

**Figure 4:**
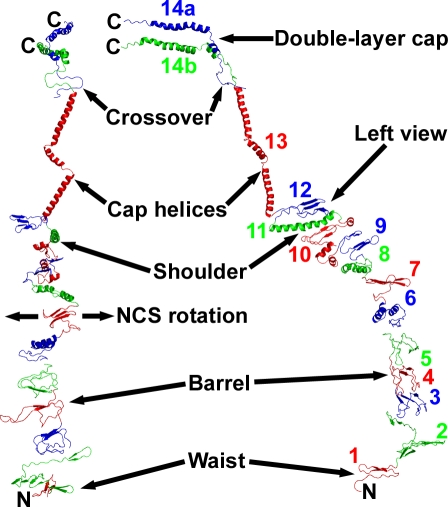
The Unique Parts of the cpMVP Model, in Two Overall Views The current cpMVP model contains 749 of the expected 873 cpMVP residues. The model is represented by ribbons. In the right part of the figure, the cpMVP model is oriented to resemble the cross-section shapes in Figures 1, S4, S5, and S6. The arrow at far right shows the approximate view direction for the left part of the figure. In the left view of the model, the symmetry-averaging direction is left-right (NCS axis is vertical, behind the page; direction of rotation around the NCS axis is marked NCS). Domain colors alternate (red-green-blue), with color transitions at residue numbers listed in Table 1. The colored domain numbers in the right part of the figure mark the domains and also show approximate viewpoints for Figure 5 (except domain 11). Both views of the model show one cpMVP chain (chain B) from the N-terminal residue Gly 3T to residue 715 just under the crossover zone of domains 14a and 14b. At the crossover (Figure 5k), the 48-fold symmetry transitions to 24-fold. Two cpMVP chains (chains A and B) are shown on their nonequivalent paths from the crossover to the C termini of domains 14a and 14b (two residue 779′s marked C). The cpMVP dimer model (PDB entry 2QZV) was completed from the unique model shown here by rotation of chain B residues 3T to 715 by one leftward increment of 48-fold NCS rotation. The cpMVP dimer model is 354 Å and 368 Å from the N termini to their corresponding inner and outer C termini. The residue numbers and locations in this model will help identify trial modification sites for engineered vaults. The two figure components were made with PyMOL [46], then combined and labeled with Adobe Photoshop.

Each half vault consists of 24 identical pairs of MVP chains A and B. Chains A and B differ only near their C termini. The unique parts of the cpMVP model (chain B and C terminus of chain A) were built into the electron density map resulting from “dot model refinement” (Figure 3). Because of nonequivalence of the C termini, the unique part of the model was assembled from 15 models of 14 domains. The stack of 15 domain models is shown in Figure 4 (see Table 1 for domain partitions; see Methods for construction details and for model validation). The cpMVP model contains 749 of the 873 residues expected for this construct, starting at residue 3T of the N-terminal cysteine tag inside the vault waist, and ending in nonequivalent residues 779 in the two C-terminal cap disks. C-terminal residues 780–861 appear to be located outside the vault, above the present model (VAK, LHR, and P. Stewart, unpublished data).

**Figure 5:**
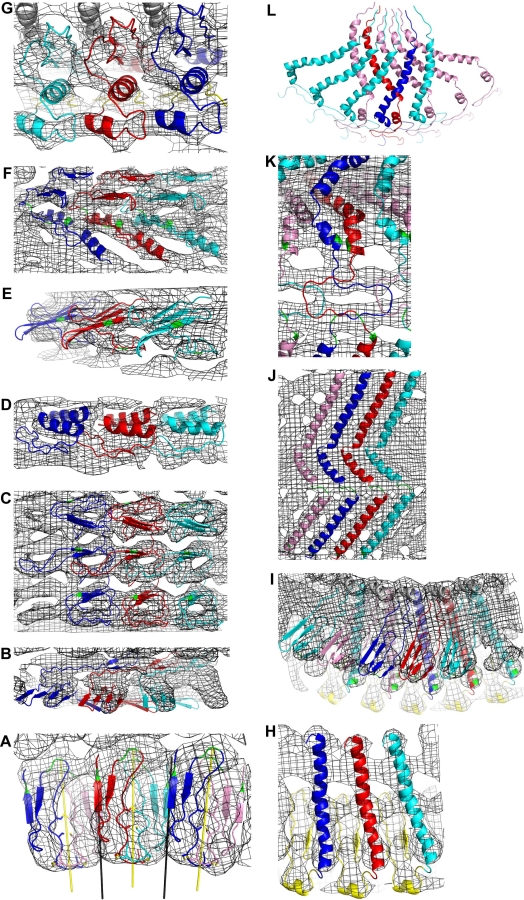
CpMVP Domain Models The cpMVP chains are shown in ribbon representation. Except as noted, chain A (leading to outer C terminus) is blue. NCS-related type A chains are cyan. Chain B (leading to inner C terminus) is red. NCS-related type B chains are pink. Residues discussed in the text are green. The *F*_observed_ electron density map is displayed as wire frame on a 2.6-Å grid. Except as noted, the viewpoints for these figures are at the approximate locations of the colored numbers in Figure 4, and “up,” “down,” “left,” “right” refer to the left part of Figure 4. **(**A) Domain 1. The viewpoint is at the red “1” in the right part of Figure 4, looking down and left from that point (into the paper). The N-terminal domains at the vault waist nestle between local (yellow) and global (black) 2-folds. Type A chains (outer C termini) are blue (top half vault) and cyan (bottom half vault). Type B chains (inner C termini) are red (top half) and pink (bottom half). The cysteines at the yellow local 2-folds disulfide bridge nonequivalent cpMVP chains in the upper and lower vault halves. Green residues are Glu 4, Glu 5, and Asp 20. Domains in the top and bottom vault halves are staggered, not stacked (see Figure 6B). (B) Domain 2. (C) Domains 3, 4, and 5, derived from the NMR substructure (PDB entry 1Y7X). The density shape nearly repeats in these domains. Green residues are tryptophans 143, 196, and 249. (D) Domain 6. (E) Domain 7. The viewpoint is at the red “7” in Figure 4, looking left (out of the paper). Green residues are prolines 367 and 381. (F) Domains 8 and 9. Green residues are prolines 420, 445, and 448. **(**G) Domain 10. The figure also shows three copies of part of domain 9 (yellow ribbon in background) and three copies of about half of domain 11 (gray helix at top). (H) Domain 11. The viewpoint is at the blue “12” in Figure 4, looking down. Domain 12 has been removed from the foreground. Three copies of domain 10 are shown as yellow ribbon in the background. The volume enclosed by two copies of domain 11, domain 10 underneath, and domain 12 above could be a lipid binding site. (I) Domain 12. The helical domain 11, and parts of domains 10 (yellow, bottom) and 13 (gray, top) are also shown. The type A chain at far right (cyan) reaches across domain 11 of chain B (red) towards a contact with chain A (blue) from two positions left. Similarly, chain B reaches across chain A to contact a type B chain (pink) two positions left. Green residues are aspartates 566, 570, and 615. (J) Domain 13. The alternating type A/type B pattern repeats left-right from what is shown. Green residues are Pro 645 (bottom) and Ala-Ala-Ala 671–673 (below center). (K) Crossover portion of domains 14a and 14b. The viewpoint is approximately at the “D” of the word “Double” in Figure 4. The crossover model reduces symmetry from 48-fold (up to residue 715), to 24-fold (residues 716 to 779). At the top of this figure, the density (at higher contour) indicated that the nonequivalent MVP chains enter the C-terminal disks in opposite directions. The upper and lower C-terminal disk models were built upside down relative to each other. Green residues are Ser 718 (bottom), Gly 720 (lower ring), and Gly 737 (center). (L) C-terminal cap disk portion of domains 14a and 14b. The view point is approximately at the “14a” mark in Figure 4, with the crossover zones at bottom. Each outer C-terminal type A chain (blue and cyan) contacts an upside down type B chain to its left, and crosses over four type B chains to its right. Each inner C-terminal type B chain (red and pink) contacts a type A chain to its right, and crosses underneath four type A chains to its left. Each panel was made with PyMOL.

**Figure 6:**
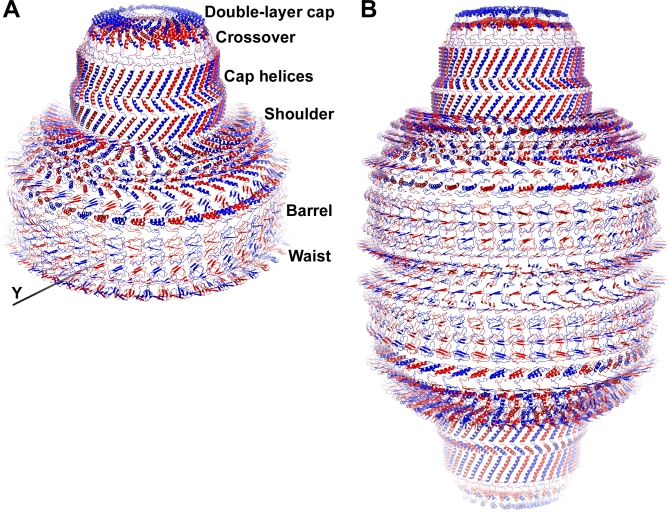
Assembly of the cpMVP Vault Shell Model (A) The asymmetric unit of the crystal contains a half vault. This half-vault model was assembled from the cpMVP dimer model (one red-blue pair) by 24-fold NCS rotation (axis marked NCS in Figures 1 and 2). The blue ribbons are type A chains (outer C termini). The red ribbons are type B chains (inner C termini). The whole vault (B) is generated from the half vault by the 2-fold rotation axis along the crystal *y* direction adjacent to the N termini at the bottom of this figure (see also Figure 5a). The many contacts between adjacent cpMVP chains may be seen in the interdigitating shapes of the domains. This figure was made with PyMOL, labeled with Photoshop. **(**B) Whole-vault model. The whole-vault model (48 cpMVP dimers) is 675 Å top to bottom, and 417 Å at the widest part of the barrel. A stack of blue domains in the upper half vault is staggered between stacks of red and blue domains in the lower half-vault. The origin of this offset is shown in Figure 5a.

The 15 domain models from three sources are shown as panels of Figure 5. Domains 3, 4, and 5 were derived from the NMR structure of domains 3 and 4 (Protein Data Bank (PDB) [18,19] entry 1Y7X [14]). Domains 1, 2, and 6–12 originated in models predicted with the ROSETTA algorithm [20–22] operating on several MVP sequence segments (see Table 1 and Methods). Domain 13 and the nonequivalent C-terminal domains 14a and 14b (see next section) were built by inserting poly-alanine segments into density, then iteratively shifting and modifying segments to pack the density with plausible topology and backbone geometry. The MVP sequence was applied to domains 13, 14a, and 14b when the other cpMVP domain models were nearly complete. As discussed in the Validation section of Methods, most domain models appear correct by the available criteria: correlation of backbone to density, plausibility of backbone geometry, and by estimation of side-chain interactions.

The MVP structure in the “crossover zone” (Figures 1, 4, and 5K) reduces the vault symmetry from 48-fold in the waist, barrel, and cap helices (residues 3T–715) to 24-fold in the C-terminal cap disks (residues 716–779). cpMVP model chains A and B become nonequivalent in the crossover zone. Assuming that identical sequences in chains A and B would result in similar structures, the crossover model was built as short A and B segments adjacent to approximate local 2-folds. The model shown in Figure 5K, when repeated 24 times and viewed at low resolution, would form the two electron density rings in the crossover zone between the two symmetries. The electron density in the two C-terminal cap disks indicated that the pairs of MVP chains enter the disks in opposite directions (Figure 5L). Reasoning as above, the C-terminal cap disk models were built upside down relative to each other. The asymmetric unit of the vault is thus a dimer of MVP molecules (model chains A and B). To complete the AB dimer model from the unique parts of the cpMVP model (Figure 4), chain B residues 3T–715 were rotated by one leftward increment of 48-fold NCS rotation to produce chain A residues 3T–715.

The asymmetric unit of the vault crystal is a half vault built by 24-fold NCS rotation of the AB dimer (blue-red pair in Figure 6A). The 417- × 417- × 675-Å^3^ whole-vault model (Figure 6B; fills density of Figure 3) is generated from the half-vault model by 2-fold rotation around the crystal *y-*axis (bottom of Figure 6A). The whole-vault model may be reconstructed from the cpMVP dimer model and rotation matrices contained in PDB entry 2QZV. Because the full model in Figure 6B (96 copies of 749 residues) is cumbersome to examine, a partially assembled cpMVP model is provided as Model S1.

## Discussion

### The Draft Model of the Vault Shell

Building an atomic model into 9-Å electron density represents crystallography at the edge of what is possible. Model building could only be attempted because the locations of the N and C termini had already been established by cryo-EM, and because the electron density of the vault shell is very thin. The “petal” shapes of collapsed vaults [3] indicated that the MVP domains stack vertically, thus limiting the volume of density to consider for each domain. That is, the sharp edges of the “petals” limit lateral excursions of the polypeptide chain, supporting the quasi-linear spoke structure that we find for MVP in the vault. In building the model, we assigned model shapes of domains into electron density shapes, resulting in what we term a draft model. We recognize the substantial uncertainties in this model, and discuss them in Text S1.

The construction of the draft model is motivated by two goals. The first is to lay a basis for further x-ray crystal studies of vaults. The next steps are crystal improvement of the vault shell and crystallization of substructures, partitioned at domain boundaries derived from our current model and sequence analysis. The substructures can be inserted into density derived from crystallography of the whole vault, as has been done for cryo-EM density of other large structures [23–25]. Such a cloning, expression, and crystallization effort could be hindered by the side-to-side interactions that build the vault (Figure 6), but these could be alleviated by residue replacements at the interaction points. The second reason to build the draft model is to guide projects of vault engineering, discussed in the following section.

### Vault Engineering

Identifying or engineering a specific property, such as metal binding, would require reasonably accurate juxtaposition of ligand atoms. We have tentatively identified some candidate metal-binding sites by the simplistic means of searching for adjacent aspartate and glutamate residues. At the local 2-fold axes between N-terminal domains (yellow bars in Figure 5A), Glu 4 and Glu 5 face Glu 4 and Glu 5 of the nonequivalent MVP in the other vault half, backed by two copies of Met 1 side chains [26]. Asp 20 in one vault half faces Asp 20 in the equivalent chain in the other vault half (across the black 2-fold bars in Figure 5A). Metal affinity at the N termini is consistent with observation of acid dissociation of vault halves [27]. The model of domain 12 (Figure 5I) reaches left to nearly bring together Asp 615 and Asp 566 (or possibly Asp 570) in domain 11 from two positions left. Thus these aspartates may be a metal affinity site.

The draft model offers ideas about the binding sites for the other vault components. Charge clusters could signify affinity sites for internal vault components. Negative charges clustered by sequence adjacency were found on the inside surface of the vault at domain 6 (Glu 342, Glu 344, Glu 346, and Glu 347). Positive charges clustered by the fold were found on the inside surface of the vault at domain 10 (Lys 506, Arg 507, His 509, Arg 511, and Arg 512). Residues 102–112 and 277–305 could not be placed in density. The site that 277–305 would occupy is slightly above the location indicated by cryo-EM analysis as the site with most binding energy for the MVP interaction domain of VPARP [15]. Atoms of 277–305 could become ordered on contact with VPARP, and this loop could be a target for insertion of a binding motif in an engineered vault.

The draft model provides a list of sequence positions likely to be loop structures where ligand-binding sequences may be inserted. Passenger proteins could then be targeted to the vault interior or exterior (analogous to [15]). The estimated domain boundaries and preliminary model may be useful for further fold predictions and fold recognitions.

### Vault Function

The draft model of the vault shell offers new conjectures about vault function. It has been suggested that vaults may interact with lipid rafts [6]. A bulk property, such as membrane binding, would be enhanced by the geometric repeating vault structure. In domains 3, 4, and 5 (as currently folded), side chains of Trp 143, Trp 196, and Trp 249 are located on an almost straight vertical line (Figure 5C). The left-right rotational repeat generates a geometric belt of membrane anchor residues around the vault barrel. The cascading energy of immersing triples of Trp side chains in a membrane could be enough to bend the membrane, or to initiate a vertical split in the vault barrel. A split vault could better contact the membrane, and could release vault contents.

An amphiphilic crevice that could bind lipid was found at the top of the vault shoulder. The inner surface of the crevice (Figure 5H) is formed by the top of domain 10, surfaces of left and right copies of domain 11, and the bottom of domain 12 (Figure 5I). The electron density for domain 12 indicates disorder, suggesting that its beta-sheet could be mobile.

The draft model hints at the origin of the striking eight-petal geometry of the collapsed vault structure [3]. How do 24 identical MVP dimers of the half vault break apart into eight identical petals? The answer may be at the top of the shoulder region. Domain 12 of each cpMVP chain overhangs two copies of domain 11 to tie together groups of three cpMVP molecules (see left panel of Figure 4 and top of shoulder in Figure 5I). This is at the base of the coiled-coil region previously thought to stabilize the vault [28]. Vaults may thus collapse into eight petals of six chains each (see Figure 9 of [3]) in part because the MVPs are tied together as threes at the top of the shoulder but twos in the barrel region.

An MVP C-terminal structure very similar to the nonequivalent C termini of this model (top of Figure 4, and Figure 5L) could be responsible for previous observations of TEP1 density [29]. The model contains two C-terminal disks built upside down relative to each other. According to this model, if TEP1 and its RNA localize to the internal surface of the inner disk, they would find similar contacts on the exterior of the outer disk. Cryo-EM analysis of various recombinant vaults containing the cpMVP construct used in this study were unable to identify a TEP1 site for lack of strong difference density [2]. However, as there are thought to be only 1–2 copies of TEP1 per vault half, it may be difficult to assign density to TEP1 in the absence of a higher-resolution structure.

These few examples of new insights into vault engineering and vault function demonstrate the potential usefulness of the draft model of the vault shell described in this paper.

## Materials and Methods

### Vaults.

The vault construct most successful for crystallography thus far was cpMVP (96 copies of 96.8 kDa; [2]). The N-terminal 12-residue sequence of cpMVP (MAGCGCPCGCGA) originated in a metal-binding motif of metallothionein. The rest of the sequence (861 residues) is the same as the rat liver MVP sequence (GenBank accession code Q62667 GI:47606697). The N-terminal tag was intended for heavy metal binding to help determine phases and thus the structure, but it instead forms disulfide links thought to rigidify the cpMVP vault and improve diffraction. cpMVP vault particles were purified as described elsewhere [5]. Further details are given in Text S2.

### Crystallization and data collection.

Crystals were grown by hanging-drop vapor diffusion. Separate reservoir and precipitant solutions decoupled the initial and destination drop conditions and were prepared as follows. The 1-ml reservoir solutions contained 0.64%–0.76% polyethylene glycol (PEG) 8000, 3% glycerol, 0.05 M Na MOPS, pH 7, 0.044 M MgCl_2_, and 0.2% n-octyl-β-D-glucopyranoside (β-OG). If a 1-mM dithiothreitol (DTT) solution was used instead of water to keep the volumes constant, the reservoir DTT concentration was about 0.8 mM. DTT seems to delay crystallization while encouraging growth of the favored C2 crystal form. The glycerol and detergent minimally affected crystallization, but they did facilitate later cryoprotection and reduce surface tension around the crystal. The volume of water (or 1 mM DTT) in the reservoir was critical to set the destination vapor pressure; one pipet was calibrated to deliver this volume. The precipitant solutions contained 0.27%–0.33% PEG 8000, 1.5% glycerol, 0.025 M Na MOPS, pH 7, 0.02 M MgCl_2_, and 0.1% β-OG. The total volumes were completed with water (or with 1 mM DTT to final concentration 0.9 mM). The precipitant mixtures were centrifuged at 10,000*g* for 3 min. The hanging drops were made by mixing 1.5-μl vault and 3-μl precipitant solutions. The air volume was initially saturated with cyclohexane (see Text S2 for further details). Crystallizations were partially protected from room vibrations by low-cost isolator platforms (Text S3). Crystals were cryoprotected and annealed by floating microdialysis (Text S4 and Figure S2). Diffraction data were collected at Advanced Light Source Beamline 8.2.2. The x-ray beam was focused at detector position (Text S5).

### Initial phasing.

Initial phases were generated by manually placing half of the cryo-EM vault electron density in the crystal lattice at a 2-fold as directed by the 13.68° β angle reported by the molecular replacement rotation function. This is the same as the tilt away from the orthogonal *z*-axis shown in the self-rotation function (Figure S3). Automated molecular replacement had been abandoned due to the inaccuracy of the translation function (see Text S6 and Figure S4). The placement and artefactual thinning operations are shown in Figure S5, and the packed phasing model is shown in Figure S6. The positive-only half-vault density map from cryo-EM (prepared for automated molecular replacement; Text S6) was scaled smaller (scale factor 0.96 applied with MAPMAN [30]), masked by MAPMASK [31], and the whole-vault center was translated to (0,0,0) with MAPROT [31,32]**.** This simplified density modification (Text S7). The density was re-masked at its new location, and the density was rotated −13.68° around the *y-*axis (and thinned, Figure S5) with MAPROT. The rotation function α and γ angles both coincided with the vault high-symmetry axis, and were ignored because the cryo-EM electron density varied little around that rotation. Phases were calculated from the density map (plus symmetry mates) with SFALL [31]. This initial near-featureless phasing model was almost centrosymmetric [33].

### Density modification.

The phase set derived from the initial model was improved by density modification by simultaneous application of NCS averaging, solvent-flattening, and histogram matching, using DM [31,34]. The cross-section in Figure 1 shows the relative locations of the crystal and NCS axes. The center of symmetry was broken by application of 48-fold NCS averaging (see Text S7; [33]). The enantiomer was assigned later during model building. The phases from the initial 48-fold average were further improved by iterative “dot model refinement” (Text S8), applying concentric 24- and 48-fold averaging to phase sets initiated from models of unassigned atoms (“dots”).

### Validation of the phasing processes.

Electron density features revealed by crystallographic means could be indirectly validated (see Text S8). The N-terminal disk inside the waist and the 48 holes at the top of the shoulder were independently observed via cryo-EM [2]. The globules of electron density (Figure 2) were spaced as though they represented backbone atoms, separated by side chains. Some of the predicted models (see below) and the NMR substructure [14] resembled shapes at their corresponding electron density. In the barrel region, a 3-fold repeat in the shape of the electron density paralleled expectation of sequence repeats (Figure 5c). The accumulated evidence indicated that the electron density was meaningful.

### Sequence analysis and ab initio model building.

The amino acid sequence of MVP has yielded some useful structural expectations. Using fold-prediction and fold-recognition algorithms, we sought models to facilitate the interpretation of the electron density map.

To initiate tertiary structure prediction for the first 400 residues of MVP, the sequence was divided at and near predicted domain boundaries. The seven N-terminal MVP repeats as represented in the PFAM protein domain database [35] were: residues 26–87, 88–141, 142–194, 195–247, 248–305, 306–355, and 356–404. For residues thought to be in the vault shoulder (approximately residues 404–650), several putative domain segments were created with sizes varying from 40–80 residues. In this region, domain boundary selection was first aided by prediction of loops using PSIPRED [36].

Ab initio models for each putative domain were generated with the HMMSTR/ROSETTA web server [20–22]. The HMMSTR/ROSETTA server divided the input sequence into short segments, searched a database for plausible fragment structures, then attempted to reassemble the fragments into a compact structure model, ignoring the NCS neighbors. The server quickly returned results by using shorter conformational searches with fewer repetitions than were used in the original ROSETTA algorithm [37], and by performing ab initio tertiary structure predictions on short segments of the chain, which are subsequently combined with a genetic algorithm [21]. The shapes and plausibilities of the ROSETTA models depended on the choices of input residue windows. Thus, we used the simplified web server version of ROSETTA for its speed in testing many residue ranges. The sequence segments chosen to construct the cpMVP model are listed in Table 1.

Both the HMMSTR/ROSETTA server and the 3-D-PSSM fold-recognition server [38] predicted several beta-sheet–rich domains in the N-terminal two-thirds of the MVP. The best 3-D-PSSM fold-recognition matches in this region included the seven-bladed beta propeller fold of Protein Data Bank (PDB; [18,19]) entry 2BBK, and beta-sheet–rich structures 1BQS and 1NLT. These fold-recognition matches did not fit well in the electron density. However, these calculations suggested that the N-terminal region contains several stacked beta-sheet–rich domains, in agreement with the observation of strong reflection intensities at 10-Å resolution, and in agreement with the NMR substructure [14].

We elaborated on the prior expectation of coiled-coil structure [28] in the 650–800 region of the MVP sequence. Residues 570–600 and 650–825 were predicted to be mostly helical using the PSIPRED secondary structure prediction method. Additionally, the 3-D-PSSM fold-recognition server predicted that these regions match well with long helices, such as those in PDB entries 1D7M, 1CUN, and 1KMI. The gapped alignment with PDB entry 1D7M, for instance, has 30% sequence identity to MVP residues 670–720 and 750–800. A high probability of helical dimer or trimer in the range of residues 680–750, was predicted using the MULTICOIL algorithm [39].

### Construction of the cpMVP model.

The cpMVP model was assembled from 15 domain models, shown as panels in Figure 5, and stacked in Figure 4. The origins and residue ranges of the individual models are listed in Table 1. The model contains 749 of the 873 residues expected for the cpMVP construct. The domain models were manually fit to a 9-Å resolution *F*_observed_ map calculated with enantiomer phases from slow reaveraging of Dot Model 6 (see Text S8), using XFIT of XtalView [40]. The map was contoured at 1.2σ and 2.6σ on a 2.6-Å grid. The domain models (backbone and β-carbon atoms) were manually bent to fit their density features. Segments were shifted to align backbone hydrogen bonds, to allow interdigitation of imagined sidechains, and to alleviate NCS collisions. Comments on specific domains are given in Text S9.

Each ROSETTA-predicted domain chosen for the cpMVP model contained a well-packed core structure, such as beta-sheets and helix, usually with dangling N and C termini. The shapes of the core features of each model were manually placed in electron-density shapes, and were arranged subject to the restraint that the dangling ends could later be manually reconnected to form a single covalent cpMVP chain. The most extreme manual interventions to ROSETTA models were applied to domain 2 (see Figure 5b and Text S9). Manual intervention at some proline residues is discussed in Text S1.

The vertical stacking of domain models was usually clear from the electron density and from the number of residues available for connections. In the shoulder region of the cpMVP model, boundaries between domains 8–11 are indistinct. The helix at the nominal boundary between domains 9 and 10 (residues 494–503, bottom foreground of Figure 5G) could be flipped left or right, resulting in shifting the top of the cpMVP model left or right relative to the bottom of the model. The helix was flipped to its current location because the flipped structure relieved strain in the backbone geometry, and substantially increased contact area between domains 9 and 10 of the same MVP chain.

### Energy minimization.

 Once the manually adjusted cpMVP model was complete, its backbone geometry was brought nearer to expectation values by torsion angle energy minimization using CNS [41], which used a hydrogen-bonding energy term [42]. CNS added side chain atoms. Some automatic rotamer choices were manually altered, and some segments were manually shifted. After each round of manual intervention in a refinement model segment, energy minimization was performed on that segment maintaining covalent connections at symmetry junctions (see Text S10). Model validation, including a score based on the side chain atoms from CNS, is discussed in Text S1.

## Supporting Information

Figure S1 Two Maps Calculated with Pseudo-Babinet-Inverse Phase Sets The figure shows results of pseudo-Babinet-inverse phase condensations from two of the tests of averaging parameters leading to Figure 2. Appearance of recognizable structure (such as helix) will not identify the true phase set at the low resolution of this analysis. Instead, we judged plausibility of structures that would result in each electron density map.
(47 KB PDF)

Figure S2 Cryoprotection-Annealing by Floating Microdialysis The vault crystals were cryoprotected (and apparently annealed) without osmotic shock by this microdialysis protocol.
(47 KB PDF)

Figure S3 Self-Rotation Function The self-rotation function indicated the orientation of the vault in the crystal.
(88 KB PDF)

Figure S4 Best Automated Molecular Replacement Result Using cryo-EM electron density, initial phasing was attempted by automated molecular replacement, but abandoned due to inaccuracy.
(63 KB PDF)

Figure S5 Manual Molecular Replacement Initial reflection phases were calculated from the manually placed and rotated cryo-EM electron density. The figure shows the main steps of this placement.
(86 KB PDF)

Figure S6 Initial Packed Phasing Model The half vault manually placed on a crystal 2-fold axis snugly packs the cell. The figure shows a section through the packed cell and the lack of phasing model for the N termini in the waist region of the vault.
(63 KB PDF)

Model S1 Partially Assembled cpMVP Model This partially-assembled cpMVP model is more convenient to examine than the full model (Figure 6B). The file contains three cpMVP dimers of the upper half vault, and N termini of the lower half vault, with chain identifiers as defined within the file.
The file is compressed with gzip. Download uncompression tools from http://www.gzip.org/. Some molecular viewer software options for the PDB file format are listed at http://www.rcsb.org/pdb/.
(396 KB GZ).

Text S1 Validation of the cpMVP Model Qualitative and quantitative validation is discussed.
(78 KB PDF)

Text S2 Details of Preparation and Crystallization of Vaults (19 KB PDF)

Text S3 Anti-Vibration Platforms This text lists suppliers, part numbers, and derivation of the part numbers for the low-cost, vibration-damping platforms used underneath the most recent vault crystallizations.
(12 KB PDF)

Text S4 Protocol for Cryoprotection-Annealing of Vault Crystals by Floating Microdialysis (71 KB PDF)

Text S5 Details of Crystal Evaluation and Collection and Processing of Diffraction Data (54 KB PDF)

Text S6 Initial Phasing of x-Ray Reflections Cryo-EM electron density was manually placed in the crystal cell to initiate the phase set.
(14 KB PDF)

Text S7 Initial Density Modification Reflection phases were improved by symmetry averaging and solvent flattening, leading to the conclusion that MVP folds into domains.
(19 KB PDF)

Text S8 “Dot Model” Density Modification Phase Refinement This text presents the detailed protocol used for further evolution of the x-ray reflection phases and of the envelope around the vault.
(79 KB PDF)

Text S9 Domain-Specific Comments on cpMVP Model Building (92 KB PDF)

Text S10 Details of Energy Minimization of the cpMVP Model (11 KB PDF)

### Accession Numbers

The 9-Å resolution cpMVP dimer model, the structure factors, and the phases used to calculate electron density maps, have been deposited in the Protein Data Bank [18,19] (http://www.rcsb.org/pdb) with accession code 2QZV. The 96-mer vault nanocapsule (Figure 6B) may be reconstructed from the cpMVP dimer using rotation matrices contained in 2QZV, for example with graphics program CHIMERA [43]. The NMR structure of domains 3 and 4 is entry 1Y7X [14]. For convenience, a partially-assembled model is available as Model S1. We again warn users of this model that its atom positions are approximate.

The GenBank (http://www.ncbi.nlm.nih.gov/Genbank) accession number for rat liver MVP sequence is Q62667.

## Abbreviations

β-OG: n-octyl-β-D-glucopyranoside detergent
σ: sigma contour level, in multiples of root mean square of the electron density values
cpMVP: major vault protein with N-terminal cysteine peptide insertion
cryo-EM: cryo-electron microscopy
DTT: dithiothreitol
MVP: major vault protein
NCS: noncrystallographic symmetry
NMR: nuclear magnetic resonance
PEG: polyethylene glycol
TEP1: telomerase associated protein 1
VPARP: vault poly(ADP-ribose)polymerase
