# Supplementary material for: Draft Crystal Structure of the Vault Shell at 9-Å Resolution
Source: PLoS Biol. 2007 Nov 27;5(11):e318. doi: 10.1371/journal.pbio.0050318 (PMC2229873; doi:10.1371/journal.pbio.0050318)
Supplement: Figure S1 — The figure shows results of pseudo-Babinet-inverse phase condensations from two of the tests of averaging parameters leading to Figure 2. Appearance of recognizable structure (such as helix) will not identify the true phase set at the low resolution of this analysis. Instead, we judged plausibility of structures that would result in each electron density map. (47 KB PDF) [file pbio.0050318.sg001.pdf]

**Figure S1. Two maps calculated with pseudo-Babinet-inverse phase sets.** During tests of initial phasing by density modification, the phase sets "condensed" into one or the other pseudo-Babinet-related solutions. This behavior differs from the Babinet flips reported for spherical viruses [1,2]. The phases for these two maps originated in application of 48-fold NCS (marked "**NCS**" in Figs. 1 and 2) to the manually-placed cryo-EM electron density (Figs. S5 and S6). The 30Å thick map slab through the cap structure is drawn in wire frame representation, and in perspective; the top of this figure shows the C-terminal disks seen from below. The horizontal red line is 100Å long. A model built using one phase set (red map), would have had 48 MVP polypeptide chains up the sides of the cap region (left and right sides of the figure), bending in to form a single C-terminal disk, with all 48 chains converging in the mushroom shape at top center. Such a disk structure would be impossible because of crowding. The pseudo-Babinet-inverse related phase set (blue map, same as in Fig. 2) resulted in a map that would have resulted from a plausible structure. The blue map was chosen to initiate "dot model" refinement. If model-building had been initiated from this blue map, the 48 MVP chains of some conformation (at far left and right in this figure; now called "cap helices" in Figs. 1, 4, "domain 13" elsewhere) would have become non-equivalent (in what is now the "crossover zone," Fig. 5k), to form two less-crowded disks (top of this figure; now called "double-layer disks"). This figure (including the scale bar) was made with XFIT of XtalView [3], RENDER of Raster3D [4], and labeled with Photoshop.

1. Chapman MS, Tsao J, Rossmann MG (1992) Ab Initio Phase Determination for Spherical Viruses: Parameter Determination for Spherical-Shell Models. Acta CrystallogA 48: 301-312.

2. Tsao J, Chapman MS, Rossmann MG (1992) Ab Initio Phase Determination for Viruses with High Symmetry: a Feasibility Study. Acta CrystallogA 48: 293-301.
3. McRee D (1999) Practical Protein Crystallography. San Diego: Academic Press.
4. Merritt EA, Bacon DJ (1997) Raster3D: photorealistic molecular graphics. MethEnzymol 277: 505-524.

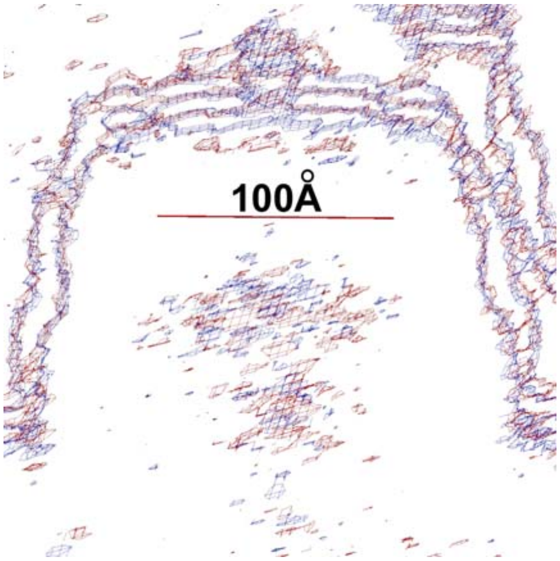
100Å
