# Supplementary material for: Draft Crystal Structure of the Vault Shell at 9-Å Resolution
Source: PLoS Biol. 2007 Nov 27;5(11):e318. doi: 10.1371/journal.pbio.0050318 (PMC2229873; doi:10.1371/journal.pbio.0050318)
Supplement: Figure S2 — The vault crystals were cryoprotected (and apparently annealed) without osmotic shock by this microdialysis protocol. (47 KB PDF) [file pbio.0050318.sg002.pdf]

Figure S2. Microdialysis cryoprotection-annealing. This method reduced osmotic shock during cryoprotection of crystals of hollow vaults. The filter membrane floats on cryoprotectant (see Text S4). The crystals were transferred *via* pipet to the top surface of the membrane. Gradual increase of glycerol and PEG concentrations at crystal position gently cryoprotected and apparently also annealed the vault crystals. This figure was made from object descriptors for the RENDER program of Raster3D [1].

1. Merritt EA, Bacon DJ (1997) Raster3D: photorealistic molecular graphics. MethEnzymol 277: 505-524.

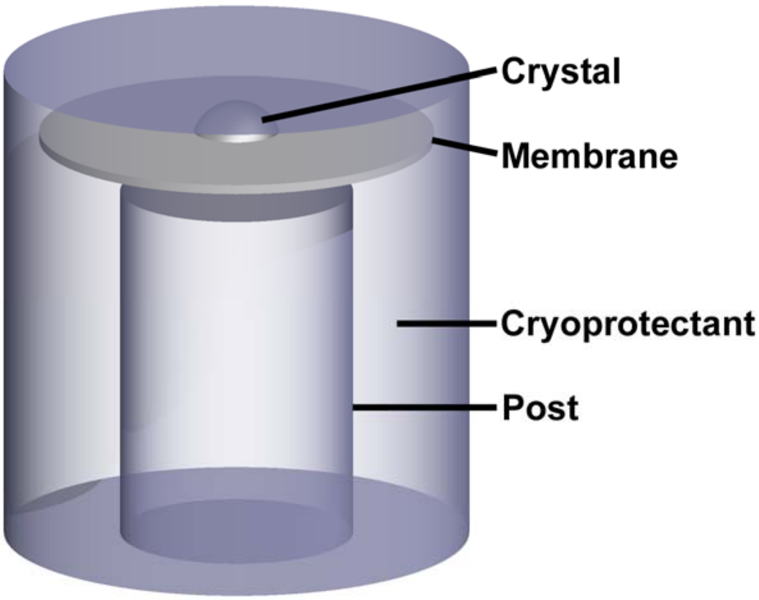
Crystal
Membrane
Cryoprotectant
Post
