# Supplementary material for: Draft Crystal Structure of the Vault Shell at 9-Å Resolution
Source: PLoS Biol. 2007 Nov 27;5(11):e318. doi: 10.1371/journal.pbio.0050318 (PMC2229873; doi:10.1371/journal.pbio.0050318)
Supplement: Figure S3 — The self-rotation function indicated the orientation of the vault in the crystal. (88 KB PDF) [file pbio.0050318.sg003.pdf]

Figure S3. Self-rotation function, 180° section. Each internal symmetry axis of the vault generates a self-rotation function peak in the direction of that axis. The function was calculated from diffraction data only and was not biased by preconceptions of vault structure. This figure (flattened representation of a convex plot) was drawn with CCP4 program MOLREP [1]. The 24 2-folds through the vault waist result in the unresolved streak extending from the **+Y** to **–Y** directions. The high-symmetry axis is tilted -13.7° around **Y** away from the orthogonal **Z** direction, and generates the peak left of center. This peak continues at the same position in other sections as an unresolved streak representing 3-fold symmetry, 4-fold, 6-fold, etc. The -13.7° tilt was applied to the cryo-EM model for manual molecular replacement (Figs. S5, S6).

1. CCP4 (1994) The CCP4 suite: programs for protein crystallography. Acta Crystallogr D Biol Crystallogr 50: 760-763.

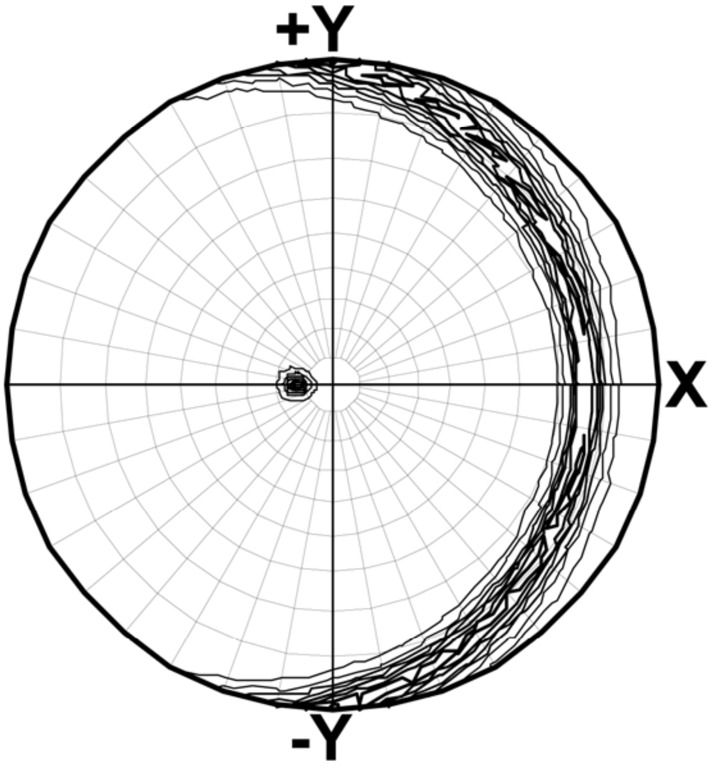
+Y
X
-Y
