# Supplementary material for: Draft Crystal Structure of the Vault Shell at 9-Å Resolution
Source: PLoS Biol. 2007 Nov 27;5(11):e318. doi: 10.1371/journal.pbio.0050318 (PMC2229873; doi:10.1371/journal.pbio.0050318)
Supplement: Figure S4 — Using cryo-EM electron density, initial phasing was attempted by automated molecular replacement, but abandoned due to inaccuracy. (63 KB PDF) [file pbio.0050318.sg004.pdf]

Figure S4. Best molecular replacement solution. The phasing model was positioned in the cell (with MAPMASK and MAPROT [1]) as instructed by MOLREP [1,2] rotation and translation results. The map is sectioned perpendicular to **Y** through the center of the vault, but not at **Y**=0. The N-termini (and other regions not shown here) are badly overlapped, while the cap and shoulder minimally contact. The NCS operators would have been un-necessarily complicated by placement of the vault center on such an arbitrary choice of crystal origin (black dots mark the crystal 2-folds along **Y** passing through two vault centers). This figure was made with MAPSLICER [1] and labeled with Adobe Photoshop.

1. CCP4 (1994) The CCP4 suite: programs for protein crystallography. Acta Crystallogr D Biol Crystallogr 50: 760-763.
2. Vagin A, Teplyakov A (1997) MOLREP: an automated program for molecular replacement. JApplCryst 30: 1022-1025.

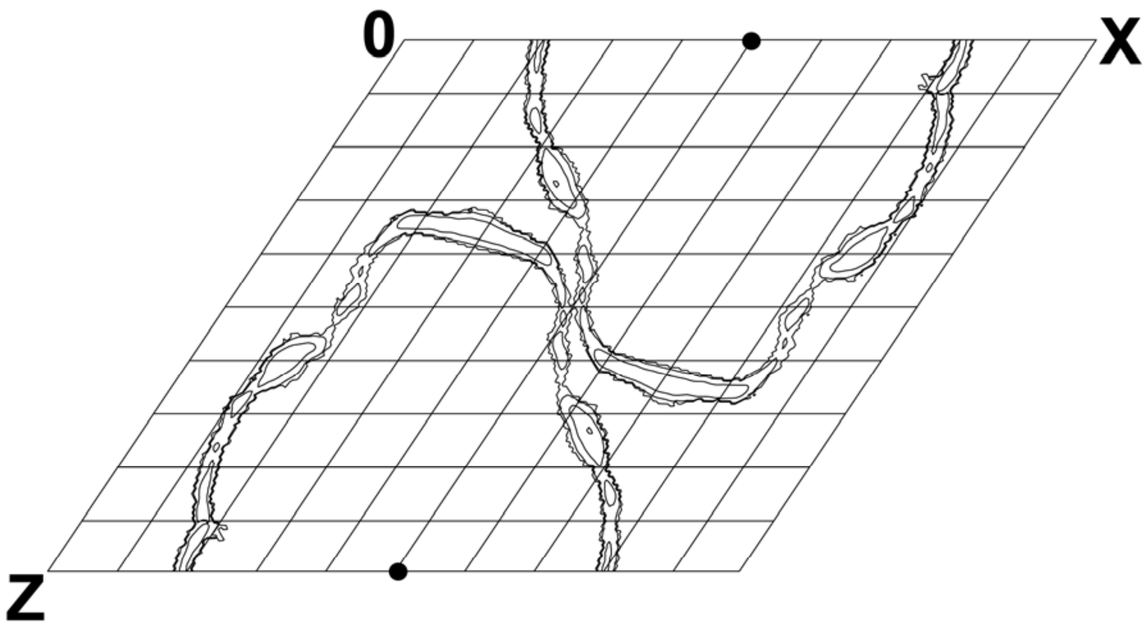
0
X
Z
