# Supplementary material for: Draft Crystal Structure of the Vault Shell at 9-Å Resolution
Source: PLoS Biol. 2007 Nov 27;5(11):e318. doi: 10.1371/journal.pbio.0050318 (PMC2229873; doi:10.1371/journal.pbio.0050318)
Supplement: Figure S5 — Initial reflection phases were calculated from the manually placed and rotated cryo-EM electron density. The figure shows the main steps of this placement. (86 KB PDF) [file pbio.0050318.sg005.pdf]

Figure S5. Manual molecular replacement. Automated molecular replacement did not accurately place the vault 2-fold at a crystal 2-fold (compare Supporting Figs. S4, S6). The vault cryo-EM electron density is shown in cross-section. **(a)** The half-vault density was scaled smaller by 0.96. The center point of the whole vault was translated to **(0,0,0)** (black dot), placing the high-symmetry vault axis on the orthogonal **Z** axis. **(b)** The density was rotated -13.7° around the **Y** axis to position the density in the crystal coordinate system. (The crystal **β** angle is 123.8° between the **X** and **Z** axes.) The crystal 2-fold along **Y** (perpendicular to the black dot at **(0,0,0)**) generated the whole vault (see Fig. S6). The density rotation program thinned the vault shell. Thickness and isotropic shrinkage of the vault were adjusted to pack the cell (see *Methods* and Text S7). The two figure components were made at the same scale with CCP4 program MAPSLICER [1], then combined and labeled with Photoshop.

1. CCP4 (1994) The CCP4 suite: programs for protein crystallography. Acta Crystallogr D Biol Crystallogr 50: 760-763.

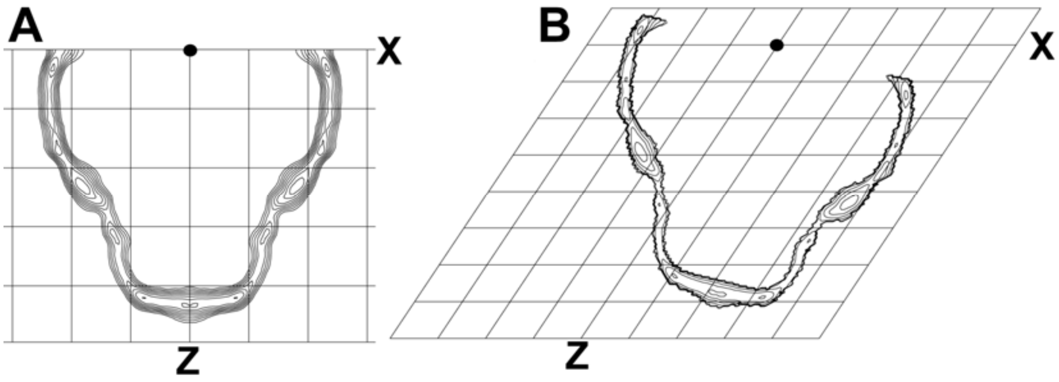
A
X
Z
B
X
Z
