# Supplementary material for: Draft Crystal Structure of the Vault Shell at 9-Å Resolution
Source: PLoS Biol. 2007 Nov 27;5(11):e318. doi: 10.1371/journal.pbio.0050318 (PMC2229873; doi:10.1371/journal.pbio.0050318)
Supplement: Figure S6 — The half vault manually placed on a crystal 2-fold axis snugly packs the cell. The figure shows a section through the packed cell and the lack of phasing model for the N termini in the waist region of the vault. (63 KB PDF) [file pbio.0050318.sg006.pdf]

Figure S6. Initial half-vault phasing model manually placed at (0,0,0). The half-vault electron density from cryo-EM was placed relative to the crystal axes as shown in Fig. S5, simplifying the NCS operators (compare to Fig. S4). The crystal 2-folds along **Y** (perpendicular to the page at the black dots) generated the whole vaults. The density rotation programs (MAPMASK and MAPROT [1]) deleted surface voxels. Consequently, there was no phasing model for the N-termini, and in this section the cap and shoulder just touch. To make this figure, the map was sectioned at **Y** = 0 (with MAPSLICER [1]). Vaults above and below (translated by variants of the centering operation **(X+1/2, Y+1/2, Z)**) pack snugly against this layer.

1. CCP4 (1994) The CCP4 suite: programs for protein crystallography. Acta Crystallogr D Biol Crystallogr 50: 760-763.

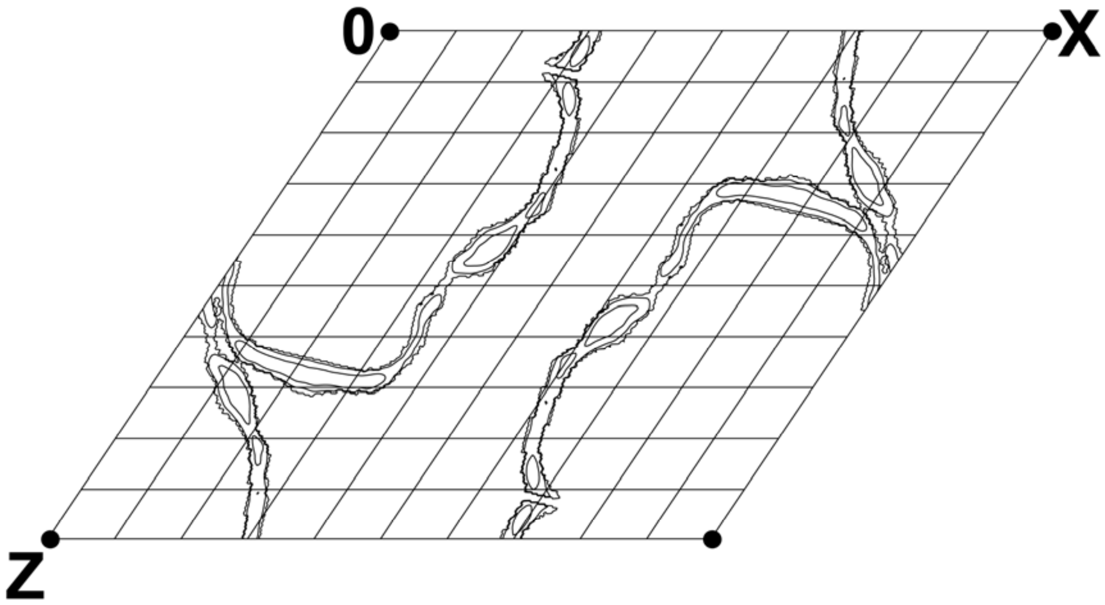
0
X
Z
