# Supplementary material for: Draft Crystal Structure of the Vault Shell at 9-Å Resolution
Source: PLoS Biol. 2007 Nov 27;5(11):e318. doi: 10.1371/journal.pbio.0050318 (PMC2229873; doi:10.1371/journal.pbio.0050318)
Supplement: Text S1 — Qualitative and quantitative validation is discussed. (78 KB PDF) [file pbio.0050318.sd002.pdf]

Text S1. Validation of the cpMVP model.

As discussed in the *Validation of Phasing Processes* section (part of Text S8), the value of the traditional R-factor is in an unfamiliar range in part because of hyper-centric intensity statistics [1]. The cpMVP model has constant "B-factor" (set arbitrarily at 50Å^2), even though some atoms occupy a fraction of the density volume, and other atoms were placed outside density. The R-factor calculated from PDB entry 2QZV (side chains limited to CB atoms) is 0.6 ($\mathbf{F}_{calc}$ from SFALL, re-scaled with RSTATS [2]), higher than the R-factor for the more graduated electron density. The R-factor is much lower for strong intensities, much higher for weak intensities.

The coordinate errors of the cpMVP model are expected to be large. Energy minimization reduced deviation from target geometry to near zero for backbone atoms. Atoms in loops placed outside density could be several Ångstroms from their average positions (assuming that the model is folded correctly). Domain 6 (Fig. 5d) provides an example of identifiable coordinate error. As a result of its initial build and energy minimization, the domain 6 model is self-consistent. The two helices stayed well-centered in density left-right (**Y** coordinate), but shifted too high in density (too negative in **Z** coordinate). A similar de-centering in domain 8 (bottom of Fig. 5f) was traced to a collision with domain 7 (Fig. 5e), and was corrected (mostly by modification of domain 7) prior to making Fig. 5f.

Some manual interventions at proline sites have indicated that the MVP sequence is aligned to the electron density. The simplified ROSETTA web server [3-5] poorly handled prolines. ROSETTA modeled the upper beta-sheet of domain 7 (Fig. 5e) with an impossible hydrogen bond to the backbone nitrogen of pro 381. Domain 7 has upper and lower electron density, with a thin connector at the point where the model requires changes of direction due to prolines 367 and 381 (as shown in Fig. 5e). Residues 406-429 of domain 8 (Fig. 5f) were predicted as straight helix, with an impossible hydrogen bond at pro 420. At the 2.6σ contour level, the density is broken in the region where this helix is disrupted by pro 420; the helix was manually disrupted accordingly. The starting models for domains 8 and 9 contained impossible hydrogen bonds to prolines 445 and 448, both predicted in beta-sheet conformation. The electron density has a thin connector between pro 445 and 448, the nominal boundary between domains 8 and 9. These proline features of the model align to features in the electron density.

The MVP sequence was applied to the C-terminal poly-alanine model only after the rest of the cpMVP model was nearly complete. The sequence numbers were extrapolated from domain 12 to the start of helix in domain 13 (Fig. 5j), resulting in naming that position residue 644. This is near the predicted start of the coiled-coil region. Proline 645 is compatible with its location at the N-terminus of the helix. Hydrophobic side chains in the coiled-coil region (domain 13) appear buried between NCS neighbors (as expected from prediction of dimer). While manually building the turn now numbered 670-673, one hydrophilic side chain was thought necessary (glu 670), and flexibility was needed in three residues (ala-ala-ala 671-673). Similarly, one sequence position at the bottom of the

crossover (Fig. 5k) was postulated to be flexible (ser 718). The poly-alanine crossover model was constructed hoping that some side chains would vanish, and now contains glycines where adjacent structural elements closely approach (glycines 720, 737). The C-terminal disks (Fig. 5l) appear to form some plausible up-down contacts. The C-terminal sequence appears to be properly assigned to model positions.

Much of the model appeared sensible by visual examination of hydrophobic burial and contacts. Visual analysis was performed during manual model adjustment, and was facilitated later by the explicit side chains built by CNS [6,7] for energy minimization. Sensibility of the all-atom model may be quantified with ERRAT and VERIFY3D (both available at nihserver.mbi.ucla.edu/SAVS/; [8-10]. Difficulty in adapting domains 3, 4, and 5 to sensible folds within density led to examination of the underlying NMR model (1Y7X, [11]). The top conformer of 1Y7X contained bad contacts and much exposed hydrophobic surface (the ERRAT score was 14). The manually rebuilt domains 3, 4, and 5 (Fig. 5c) received an ERRAT score of 52, with part of domain 5 receiving by far the worst ERRAT score of the cpMVP model. For a dissected model, the ERRAT algorithm reported scores of 43 for domains 3 and 4, 76 for domain 5. The hydrophobic linker between domains 4 and 5 was manually twisted to minimize implausibility. No manual rebuilding solution has yet been found to flip asp 255 out of the domain 5 hydrophobic core. The ERRAT score is 73 for the entire cpMVP dimer model, including suspect components. This score, being less than the minimum of 90 expected for well-refined, high-resolution structures, indicates that considerable model errors remain, which will require data at higher resolution to correct.

1. Douglas AS, Woolfson MM (1954) The maximum probable value of the reliability index for a hypercentric structure. Acta Crystallog 7: 517.
2. CCP4 (1994) The CCP4 suite: programs for protein crystallography. Acta Crystallogr D Biol Crystallogr 50: 760-763.
3. Bystroff C, Thorsson V, Baker D (2000) HMMSTR: a hidden Markov model for local sequence-structure correlations in proteins. J Mol Biol 301: 173-190.
4. Bystroff C, Shao Y (2002) Fully automated ab initio protein structure prediction using I-SITES, HMMSTR and ROSETTA. Bioinformatics 18 Suppl 1: S54-61.
5. Rohl CA, Strauss CE, Misura KM, Baker D (2004) Protein structure prediction using Rosetta. Methods Enzymol 383: 66-93.
6. Brunger AT, Adams PD, Clore GM, DeLano WL, Gros P, et al. (1998) Crystallography & NMR system: A new software suite for macromolecular structure determination. Acta Crystallogr D Biol Crystallogr 54: 905-921.
7. Fabiola F, Bertram R, Korostelev A, Chapman MS (2002) An improved hydrogen bond potential: impact on medium resolution protein structures. Protein Sci 11: 1415-1423.
8. Colovos C, Yeates TO (1993) Verification of protein structures: patterns of nonbonded atomic interactions. Protein Sci 2: 1511-1519.
9. Bowie JU, Luthy R, Eisenberg D (1991) A method to identify protein sequences that fold into a known three-dimensional structure. Science 253: 164-170.
10. Luthy R, Bowie JU, Eisenberg D (1992) Assessment of protein models with three-dimensional profiles. Nature 356: 83-85.
11. Kozlov G, Vavelyuk O, Minailiuc O, Banville D, Gehring K, et al. (2006) Solution structure of a two-repeat fragment of major vault protein. J Mol Biol 356: 444-452.
