# Supplementary material for: Draft Crystal Structure of the Vault Shell at 9-Å Resolution
Source: PLoS Biol. 2007 Nov 27;5(11):e318. doi: 10.1371/journal.pbio.0050318 (PMC2229873; doi:10.1371/journal.pbio.0050318)
Supplement: Text S2 — (19 KB PDF) [file pbio.0050318.sd003.pdf]

Text S2. Preparation and crystallization of vaults.

Vaults. Crystals were grown of complete vaults purified from rat liver, and of several recombinant constructs expressed *via* baculovirus in SF9 moth cells. The rat liver and cpMVP vaults were purified as described previously [1,2]. The cpMVP crystals used here resulted from vaults in the 40% sucrose layer. Crystals of lesser quality grew from vaults in the 45% layer. To exchange the buffer in and around the vaults, 1 ml of the 40% layer was diluted in 7 ml 0.05M tris(hydroxymethyl) aminomethane hydrochloride pH 7.4, 0.075 M NaCl, 0.5 mM $MgCl_2$, then pelleted by centrifugation at 100000 *g* for 2 hours. The pellet was re-suspended in a volume (typically 0.1-0.2 ml) of 0.02 M Na morpholine ethane sulfonate (MES) buffer at pH 6.5 that would exceed the target vault concentration of 2.7 mg/ml.

Better vault crystals have grown as the cpMVP preparations aged, as though this construct of MVP anneals by air oxidation of the cysteines at the N-termini. The two best crystals thus far (resulting in this publication) grew from the last microliters of a 5-week-old preparation. Unlike the previous best crystals, these grew with the reductant option of the protocol, as though disulfide re-annealing is beneficial.

Crystallization. Two conditions for vault crystallization were developed to the point of good-looking crystals. The later protocol with 2-methyl-2,4-pentanediol as precipitant has not led to diffraction, but provided important insights about aggregation that were applied to the earlier polyethylene glycol (PEG) protocol. The PEG protocol mostly

evolved while working with the cpMVP construct, but appears near optimal for crystallization of vaults purified from rat liver.

With the goal of a second easily-cryoprotected vault crystal with low surface tension, 2-methyl-2,4-pentanediol (MPD) was explored as precipitant in a vapor-diffusion protocol. Dilute MPD was expected to have little influence on water vapor pressure. NaCl was used in the reservoir to set the destination drop condition. The reservoir solutions were 1ml of 5% MPD, 0.05 M Na 3-(N-morpholino) propane sulfonate (MOPS) pH 7, scanning 0.070 to 0.085 M NaCl. Each reservoir was covered with 0.2 ml of 1:1 mineral and silicone oils [3] to slow the drop trajectories towards the endpoints. The precipitant solutions to add to the vaults solution contained: 5% MPD, 0.04 M $MgCl_2$, 0.05 M Na MOPS pH 7. More than enough vaults for a row of drops were mixed with an equal volume of precipitant, and centrifuged at 3000 g for 3 minutes. For example 5 µl 2.5 mg/ml vaults were mixed with 5 µl precipitant for 4 drops of 2 µl each. Pipetting from the top of the vault solution, almost all the particulates were left behind in the 2 µl excess volume. The drops were pipetted onto siliconized glass coverslips, and inverted over the reservoirs. Crystals grew in several weeks at about 18-21°C.

Unlike the MPD condition, centrifugation of a vaults-PEG mixture removed all vaults from solution. This meant that aggregation was immediate. The PEG precipitant mixtures added to the vaults were therefore dilute. During this work, some virtues of drop dilution were published [4].

The cpMVP crystals used for structure determination were grown by the following vapor-diffusion protocol using polyethylene glycol as precipitant. The reservoir and precipitant solutions are specified in the main text.

The vaults were prepared for crystallization as follows: The optimum vault concentration was about 2.7 mg/ml. Most cpMVP preparations (5 to 11 mg/ml) were diluted with 0.02M Na MES pH 6.5 to the target 2.7 mg/ml. Dilution with this buffer maintained consistent water vapor pressure across the batches of vaults. The vaults were centrifuged at 3000 *g* for 3 minutes. Excess vault solution was prepared to avoid transfer of large particles from the bottom of the centrifuge tube to the drops. For example, for twelve 1.5 µl drops, 7.7 µl of 7.36 mg/ml vaults were diluted with 13.3 µl of 0.02 M Na MES pH 6.5, then centrifuged.

Each vapor diffusion crystallization chamber was individually assembled in a choreographed sequence to minimize evaporation and to reduce aggregation of vaults. 3 µl precipitant were added to a 1.5 µl vault drop on a siliconized coverslip, and with the same pipet tip, 3 µl were drawn in and out three times in about two seconds. This mixing was thought to shear vault aggregates for a more monomeric starting condition. Pipetting in and out also reduced the number of aggregated vaults seen on an EM grid. The coverslip was then inverted, 40µl cyclohexane (held in a positive-displacement pipet) were added onto the reservoir, and the coverslip was pressed onto the grease seal. Addition of cyclohexane vapor improved resolution from about 100-40Å to 27Å, and

increased the volume of each single domain within each crystal. Crystals became visible in about a week, but growth should be left undisturbed for at least 2 weeks at about 20°C.

1. Kong LB, Siva AC, Rome LH, Stewart PL (1999) Structure of the vault, a ubiquitous cellular component. Structure 7: 371-379.
2. Stephen AG, Raval-Fernandes S, Huynh T, Torres M, Kickhoefer VA, et al. (2001) Assembly of vault-like particles in insect cells expressing only the major vault protein. J Biol Chem 276: 23217-23220.
3. Chayen NE (1997) The role of oil in macromolecular crystallization. Structure 5: 1269-1274.
4. Dunlop KV, Hazes B (2003) When less is more: a more efficient vapour-diffusion protocol. Acta Crystallogr D Biol Crystallogr 59: 1797-1800.
