# Supplementary material for: Draft Crystal Structure of the Vault Shell at 9-Å Resolution
Source: PLoS Biol. 2007 Nov 27;5(11):e318. doi: 10.1371/journal.pbio.0050318 (PMC2229873; doi:10.1371/journal.pbio.0050318)
Supplement: Text S3 — This text lists suppliers, part numbers, and derivation of the part numbers for the low-cost, vibration-damping platforms used underneath the most recent vault crystallizations. (12 KB PDF) [file pbio.0050318.sd004.pdf]

Text S3. Suppression of vibrations. Low-cost platforms isolated the hanging drops of the most recent vault crystallizations from high-frequency vibrations transmitted through the building. Intermittent high-frequency vibrations can resonate with the hanging drops and perturb crystallization. A particle-board bookshelf compresses four soft bumpers placed under the corners. To isolate crystallization plates from shocks due to placing additional plates on the same platform, the top surface of the platform was covered with a soft foam.

The hemispherical “adhesive-backed load-rated soft bumpers” were manufactured by Sorbothane (www.sorbothane.com), but their minimum order prohibited prototyping. Components for the anti-jiggle platforms were purchased from McMaster-Carr (www.mcmaster.com). The bumper diameter and hardness were chosen for the estimated load per bumper; the bumpers should compress 20-30% under load. Some particle-board shelves were weighed to derive a density, and this density was used to estimate the weight of a platform that would fit on a shelf inside a cabinet in a temperature-controlled room. Filled 24-well crystallization plates were weighed to derive a load per plate. Assuming 5 stacks of 5 plates stored on a 10 by 36 inch shelf (McMaster 50385T3), the estimated load per bumper corresponds to the 1.25 inch hemisphere, Durometer 30 (Sorbothane 0510121; McMaster 8215K2). A heavier load estimate would correspond to a larger bumper (Sorbothane 0510141; McMaster 8215K3). The top surface of the platform is covered with “non-slip polyester/vinyl perforated foam” (similar to an anti-slip pad intended for dishes in a kitchen), 36 inches wide (McMaster 85695K4), trimmed to fit.
