# Supplementary material for: Draft Crystal Structure of the Vault Shell at 9-Å Resolution
Source: PLoS Biol. 2007 Nov 27;5(11):e318. doi: 10.1371/journal.pbio.0050318 (PMC2229873; doi:10.1371/journal.pbio.0050318)
Supplement: Text S4 — (71 KB PDF) [file pbio.0050318.sd005.pdf]

Text S4. Cryoprotection-annealing by microdialysis. The diffraction from the vault crystals was further improved by a microdialysis protocol (Fig. S2). Conventional means of cryoprotection destroyed the vault crystals; the MPD form crystals visibly collapsed. The $5x10^7$ $Å^3$ enclosed volume of each vault shell [1] must be cryoprotected; cryoprotection of the outside surface of the crystal is not sufficient [2]. By microdialysis, glycerol diffused through the vault pores without bulk osmotic shock. This is a variant of a heavy-metal soak protocol used previously [3]. The solution for transfer of crystals from growth drop to microdialyzer contained: 0.8% PEG 8000, 3% glycerol, 0.05 M Na MOPS pH 7, 0.04 M $MgCl_2$, 0.2% β-OG. The cryoprotectant was usually 3-4% PEG 8000, 30% glycerol, 0.05 M Na MOPS pH 7, 0.04 M $MgCl_2$, 0.2% β-OG. The dialyzer was a 13 mm Millipore type VSWP membrane, floating on 1.84ml of cryoprotectant, hydrophobic (shiny) side up. The container was a Hampton Research "Cryschem" 24-well plate, chosen because the sitting-drop post provides mechanical stabilization. About 10 µl of transfer solution was added to the crystals in their growth drop, a vault crystal was sucked into the same pipet tip and transferred to top center of the dialyzer membrane. The opening in the pipet tip must be larger than the crystal. The crystals used for data collection were dialyzed overnight, but more recent crystals seemed cryoprotected after about 3 hours of dialysis. The dialyzing crystals may be safely hand-carried between rooms on a tray weighted with some lead. The glycerol cryoprotectant rarely floods the dialyzer. MPD soaks are much more fragile because the MPD more strongly wets the top surface. The dialyzing crystals are routinely difficult to see until they gain index of refraction. This dialysis protocol appears to also anneal the PEG-form vault crystals (for a review of annealing, see [4]).

1. Kong LB, Siva AC, Rome LH, Stewart PL (1999) Structure of the vault, a ubiquitous cellular component. Structure 7: 371-379.
2. Pflugrath JW (2004) Macromolecular cryocrystallography--methods for cooling and mounting protein crystals at cryogenic temperatures. Methods 34: 415-423.
3. Anderson DH (1986) The Use of a Multiwire Area Detector Diffractometer,
the Crystal Structure of the Compound I Decay Product of Cytochrome c Peroxidase, and a Crystallographic Study of Phospholipase A2. San Diego: University of California.
4. Heras B, Martin JL (2005) Post-crystallization treatments for improving diffraction quality of protein crystals. Acta Crystallogr D Biol Crystallogr 61: 1173-1180.
