# Supplementary material for: Draft Crystal Structure of the Vault Shell at 9-Å Resolution
Source: PLoS Biol. 2007 Nov 27;5(11):e318. doi: 10.1371/journal.pbio.0050318 (PMC2229873; doi:10.1371/journal.pbio.0050318)
Supplement: Text S5 — (54 KB PDF) [file pbio.0050318.sd006.pdf]

Text S5. Crystal evaluation, and collection and processing of diffraction data. The dialysis-cryoprotected crystals were flash-cooled [1], using a nitrogen stream at about 105K. Using our in-house X-ray equipment for the vault crystals, we can observe diffraction strength, but we cannot collect diffraction data. In-house, the best of the **C2** form crystals resulted in articulated but unresolved diffraction lunes. Only a few percent of the tested vault crystals have produced useable diffraction.

The diffraction data used here were collected at Advanced Light Source Beamline 8.2.2 at Lawrence Berkeley National Laboratory. With the active surface of the ADSC Q315 detector set 700 mm from the crystal position, the x-ray beam was focused on the protective cover of the detector. A bellows helium chamber with mylar windows minimized absorption and scatter by air of the diffracted rays. With the spot spacing maximized by minimized spot size, the wavelength was reduced to 1.08 Å. This wavelength maximized flux, while reducing radiation damage to the crystal relative to the previous practice of spacing reflections with a longer wavelength. Also, this modification increased the illuminated volume of crystal, and thus increased the diffracted intensities. The rows of closely-spaced reflections from a vault crystal could be mis-indexed if the beam center parameters were incorrect. Prior to vault crystal data collection, diffraction images from a tetragonal lysozyme crystal were recorded and used to determine camera parameters by multi-image indexing and refinement (see next). The 1° rotation per vault diffraction image would have overlapped reflections if the diffraction were more isotropic. The oscillation angle will be reduced if future crystals improve even slightly. Diffraction intensities were measured at 10-second exposure time. Few intensities

overloaded the detector, possibly because the hollow vault does not produce giant Fourier terms peaking at particle center.

Data processing for the two crystals was tailored to the diffraction characteristics. The diffraction was indexed with MOSFLM [2] using 7-9 images evenly spaced over 180° rotation. No single diffraction image contained enough information for Fourier indexing [3]. The best indexing of the many possibilities was: **C2** symmetry with cell parameters **a**=631.5 Å, **b**=464.7 Å, **c**=584.6 Å, **β**=123.8°. Intensities were extracted using MOSFLM, scaled with SCALA [4,5] and converted to structure factors with TRUNCATE [5]. The intensity and E-value distributions were visibly perturbed (the intensities alternated from large to near-zero, and diffraction spikes extended as far as 5 Å resolution). The E-value limit was shut off in SCALA (maximum E-value within the processed resolution range was 22), and the "truncation" protocol was shut off in TRUNCATE ("truncation" increases **F** for zero and negative intensity values).

The data sets from the two crystals were kept separate because of small differences in cell parameters and structure factors. All operations up to the point of Fig. 2 (see *Density modification*, Text S7) were performed in parallel for the two data sets, with indistinguishable results. The data set used beyond Fig. 2 was processed from 617249 observations of 101683 reflections. The overall R-factor ("R_{sym}") was 0.22 for the reflections between 190 and 9 Å. The R-factor rose to a plateau of about 0.45 around 11 Å, then spiked to 0.63 in the 9.49-9 Å shell (where average (intensity/error estimate) was

1.2). Completeness was 98%, and multiplicity was 6.1 (95% and 5.3 in the last shell).

Data beyond 9 Å resolution were judged not useable.

1. Pflugrath JW (2004) Macromolecular cryocrystallography--methods for cooling and mounting protein crystals at cryogenic temperatures. Methods 34: 415-423.
2. Powell HR (1999) The Rossmann Fourier autoindexing algorithm in MOSFLM. Acta Crystallog D 55: 1690-1695.
3. Steller I, Bolotovsky R, Rossmann MG (1997) An Algorithm for Automatic Indexing of Oscillation Images using Fourier Analysis. JApplCryst 30: 1036-1040.
4. Evans PR (1997) Scala. Joint CCP4 and ESF-EACBM Newsletter on Protein Crystallography 33: 22-24.
5. CCP4 (1994) The CCP4 suite: programs for protein crystallography. Acta Crystallogr D Biol Crystallogr 50: 760-763.
