# Supplementary material for: Draft Crystal Structure of the Vault Shell at 9-Å Resolution
Source: PLoS Biol. 2007 Nov 27;5(11):e318. doi: 10.1371/journal.pbio.0050318 (PMC2229873; doi:10.1371/journal.pbio.0050318)
Supplement: Text S6 — Cryo-EM electron density was manually placed in the crystal cell to initiate the phase set. (14 KB PDF) [file pbio.0050318.sd007.pdf]

Text S6. Initial phasing. Automated molecular replacement was attempted for initial phasing, but was abandoned because of the inaccuracy of the translation function. The search model was derived from a cryo-EM electron-density map at a nominal 21 Å resolution. This map was a predecessor of that reported by Mikyas, et al [1]. Negative electron densities resulting from ringing were set to zero (to flatten the solvent region), and positive densities were multiplied by 10 only to increase legibility of the map files (in retrospect un-necessary). To produce the half-vault search model, the whole-vault model in its 815 Å cube was cut in half at the N-terminal waist region ("**48N**" in Fig. 1; [1]). To remove some cross-vectors between the half-vaults, a 40 Å thick slab of zero density was inserted at the cut (also un-necessary). These operations were performed with CCP4 [2] programs EXTEND and ZEROED. Automated molecular replacement was run with MOLREP [2,3], which calculated a rotation function integration radius of 255 Å. Initial phasing models were produced by applying the rotation and translation parameters from MOLREP with MAPMASK [2] and MAPROT [2,4]. MAPROT deleted surface voxels during rotation of the density, thinning the vault shell. The cell could be packed with this thinned model by isotropic shrinkage of the initial model (0.96 scale factor applied to the starting map cell parameters using MAPMAN [5]). The magnification parameter for the cryo-EM analysis of vaults was calibrated by comparison to a catalase crystal, with an estimated error of 5% (same as in [6]). The rotation function was insensitive to the shrinkage parameter in the tested range of 1.0 to 0.95. In retrospect, the translation function may have not produced a clear solution because MOLREP used the original thick model (with excessive density contrast) and always overlapped density. This effort failed to accurately align the vault N-terminal 2-fold to a crystal 2-fold axis with good

contacts and no collisions. The best result is shown in Supporting Fig. S4; the N-terminal collision may be seen by comparison with Fig. S6. The solution in Fig. S4 produced a rotation function peak of 6.28 times background, and a translation peak of 2.11 times background, similar to other solutions. The useful results from automated molecular replacement were that the crystal vault is 4% smaller than the cryo-EM solution vault, and that one vault 2-fold overlaps the crystal 2-fold. Thus the vault barrel shape is preserved in the crystal.

1. Mikyas Y, Makabi M, Raval-Fernandes S, Harrington L, Kickhoefer VA, et al. (2004) Cryoelectron microscopy imaging of recombinant and tissue derived vaults: localization of the MVP N termini and VPARP. J Mol Biol 344: 91-105.
2. CCP4 (1994) The CCP4 suite: programs for protein crystallography. Acta Crystallogr D Biol Crystallogr 50: 760-763.
3. Vagin A, Teplyakov A (1997) MOLREP: an automated program for molecular replacement. JApplCryst 30: 1022-1025.
4. Stein PE, Boodhoo A, Armstrong GD, Cockle SA, Klein MH, et al. (1994) The crystal structure of pertussis toxin. Structure 2: 45-57.
5. Kleywegt GJ, Jones TA (1996) xdlMAPMAN and xdlDATAMAN - Programs for reformatting, analysis and manipulation of biomacromolecular electron-density maps and reflection data sets. Acta CrystallogD 52: 826-828.
6. Chiu CY, Cary RB, Chen DJ, Peterson SR, Stewart PL (1998) Cryo-EM imaging of the catalytic subunit of the DNA-dependent protein kinase. J Mol Biol 284: 1075-1081.
