# Supplementary material for: Draft Crystal Structure of the Vault Shell at 9-Å Resolution
Source: PLoS Biol. 2007 Nov 27;5(11):e318. doi: 10.1371/journal.pbio.0050318 (PMC2229873; doi:10.1371/journal.pbio.0050318)
Supplement: Text S7 — Reflection phases were improved by symmetry averaging and solvent flattening, leading to the conclusion that MVP folds into domains. (19 KB PDF) [file pbio.0050318.sd008.pdf]

Text S7. Density Modification.

Phasing parameters were tested by a variant of the radius tests performed earlier for spherical viruses [1,2]. At low resolution, a spherical virus may be modeled as a hollow shell of electron density. The choice of inner and outer radius parameters impacts the course of phase "condensation" during density modification. For vaults, two model thicknesses were tested: One was scaled by 0.96 to fit the cell, and artefactually thinned during rotation by MAPROT ([3]; Figs. S5 and S6). To pack the cell with a thicker model, the model was scaled by 0.95, and the mask around the model at **(0,0,0)** was expanded (with MAMA [4]) prior to -13.68° rotation with MAPROT. The averaging mask around this thicker phasing model overlapped the lattice contacts.

Manual placement of the high-symmetry axis exactly perpendicular to **Y** exactly at **(0,0,0)** (*Methods* section in main text) simplified the non-crystallographic symmetry operations by eliminating translational components. The **n***th* 48-fold NCS matrix was generated by the product of three rotation matrices: +13.68° rotation around **Y** to bring the high-symmetry axis onto the orthogonal **Z** direction; 360**n**°/48 around **Z**; then -13.68° around **Y** to reposition the density in the crystal cell. This matrix-generator by Robert Grothe was the only program written specifically for the vaults project.

Several averaging schemes were tested, with solvent flattening and histogram matching applied in each test. Application of 8-fold rotational NCS revealed 48-fold periodicities in the shoulder region of the map, thus confirming that the vault contains 96 copies of MVP [5]. 16- and 24-folds were also tested. The thin model was averaged inside a thin

mask, and also allowed to grow into a thicker mask (expanded with MAMA after rotation of the density using MAPROT). The automatic re-masking algorithm of DM usually added small detached artefactual density peaks to the mask, but in one test added a disk at the N-terminal waist region inside the vault (marked "**48N**" in Fig. 1). This disk was also observed *via* cryo-EM [5]. The N-terminal mask used for further averaging was produced by inserting some arginine molecules into the disk discovered by DM (using XFIT of XtalView [6]). Applying 48-fold NCS to the arginines (using PDBSET [3]) filled the disk of density. An N-terminal mask was drawn around the artificial atoms by NCSMASK [3], and was appended to the existing cryo-EM derived mask with MAMA. N-termini were not added to the cryo-EM phasing model (Supporting Fig. S6). Masks completed with the N-terminal disk were used in subsequent averaging tests.

Two distinct map types resulted from phase "condensations" in the NCS tests (see Fig. S1), distinct from the "condensation" behavior as first reported for spherical viruses [1,2]. The two map types were pseudo-Babinet related. Unlike a true Babinet inversion by 180° phase flips, only a subset of the phases flipped, keeping the density inside the mask, but altering interpretation of the map. The two phase set types became anti-correlated around 16 Å resolution. One "condensed" phase set was chosen for plausibility of the model that could be built into the map (see text for Supporting Fig. S1). In retrospect, the un-crowded two-layer C-terminal cap disks of the more plausible phase set had also resulted from application of solvent-flattening alone. In the map with a two-layer cap, the appearance of the N-termini (leading eventually to Fig. 5a) was thought to be consistent

with the mass spectrum result that the cpMVP construct is entirely disulfide-linked dimers (Joseph Loo, personal communication).

The map used to initiate "dot model refinement" (see Text S8) resulted from applying 48-fold NCS averaging to the thin initial model, allowing the density to grow into a thicker mask (1 voxel on each surface), and into the previously empty N-terminal extension mask. The starting resolution was 30 Å, extending to the resolution limit in 100 cycles. The resultant map (Fig. 2) was much more detailed than the featureless cryo-EM starting density.

In the above use of DM, the initial figure of merit weights were an arbitrary small number (0.2), and the "combine nocombine" instruction to DM allowed the phases to change drastically during density modification. This initial averaging was repeated much later with SIGMAA-weighting [7] and a corrected version of DM (see Text S8), resulting in a very similar map.

1. Chapman MS, Tsao J, Rossmann MG (1992) Ab Initio Phase Determination for Spherical Viruses: Parameter Determination for Spherical-Shell Models. Acta CrystallogA 48: 301-312.
2. Tsao J, Chapman MS, Rossmann MG (1992) Ab Initio Phase Determination for Viruses with High Symmetry: a Feasibility Study. Acta CrystallogA 48: 293-301.
3. CCP4 (1994) The CCP4 suite: programs for protein crystallography. Acta Crystallogr D Biol Crystallogr 50: 760-763.
4. Kleywegt GJ, Jones TA (1999) Software for handling macromolecular envelopes. Acta CrystallogD 55: 941-944.
5. Mikyas Y, Makabi M, Raval-Fernandes S, Harrington L, Kickhoefer VA, et al. (2004) Cryoelectron microscopy imaging of recombinant and tissue derived vaults: localization of the MVP N termini and VPARP. J Mol Biol 344: 91-105.
6. McRee D (1999) Practical Protein Crystallography. San Diego: Academic Press.

7. Read RJ (1986) Improved Fourier coefficients for maps using phases from partial structures with errors. Acta Crystallog A 42: 140-149.
