# Supplementary material for: Draft Crystal Structure of the Vault Shell at 9-Å Resolution
Source: PLoS Biol. 2007 Nov 27;5(11):e318. doi: 10.1371/journal.pbio.0050318 (PMC2229873; doi:10.1371/journal.pbio.0050318)
Supplement: Text S8 — This text presents the detailed protocol used for further evolution of the x-ray reflection phases and of the envelope around the vault. (79 KB PDF) [file pbio.0050318.sd009.pdf]

Text S8. “Dot model” phase refinement. “Dot models” were used to further evolve the masks and phases away from those derived from the cryo-EM start. The use of un-assigned atoms for phase improvement is very old [1], is used today for model building in ARP/wARP [2,3], and also in some direct phasing programs. Iterative dot model refinement was implemented at low resolution as follows. At the conclusion of the 48-fold NCS average (Text S7), the cpMVP density had separated into domains. One copy of each density block was chosen near **Y**=0 but far enough from lattice contacts to be representative, and its density was flooded with water molecules (“dots”) using MAPMAN [4], MAMA [5], and FLOOD [6]. Density block partitions (chosen for convenience) are shown in the cross-section Fig. 1. Each block of dots was manually edited (with XFIT of XtalView [7]) to remove overlaps with blocks above, below, left and right (directions are relative to left part of Fig. 4). Dots outside strong density were deleted. Where the local density differed from the consensus shape of adjoining NCS copies, the dots were edited to follow the consensus. To construct the half-vault phasing model, NCS operations were applied to the dot models with PDBSET [8]: 24-fold symmetry for the C-terminal cap structure (density block 11, “crossover” and “cap disks” in Figs. 1 and 4), and 48-fold for the rest. The 24- and 48-fold NCS axes were co-axial; Figs. 1 and 2 each show one “**NCS**” line. These sets of 24 and 48 NCS matrices were generated by a program and were not further refined. Phases were calculated from the half-vault dot model with SFALL, calculated and observed structure factors were scaled with RSTATS and starting weights were computed with SIGMAA ([9]; all three programs from [8]).

New masks were generated for each round of dot model refinement. Two multimer-masks were drawn around the dots with NCSMASK [8] and used for NCS averaging: a small component for the 24-fold symmetric cap components, and a much larger component for the 48-fold remainder. For most of this iterative process, the mask surfaces were 5 Å away from dots, to allow the density to reshape for the next dot model. The solvent masks were built by appending the two multimer-masks (with MAMA) and applying crystal symmetry (with MAPMASK [8]).

Symmetry averaging with solvent flattening and histogram matching was performed using DM [8,10] for 100 cycles. For the next round of density modification, dots were deleted or manually shifted into newly added density, and new masks were generated. An error resulting in a coarse grid in DM was corrected during work on the fifth dot model. The dot model refinement process seems to have converged after the fifth dot model. Dot Models 5 and 6 were terminated at the uppermost green line in Fig. 1, leaving holes in the C-terminal cap disks. No real atom could be located beyond the termini of the Dot Models (due to crowding), and the density there was thought to be noise.

The above dot model refinement effort resulted in revising the masks, without revealing detail within. Phases from Dot Models 5 and 6 were re-averaged, starting at 40 Å resolution, extending to 9 Å resolution in 500 and 600 cycles respectively, again with the "combine nocombine" instruction to DM. In this slow phase extension and annealing, the highest resolution phase probability distributions shifted and clarified the map within the envelopes. The re-averaged map from Dot Model 6 (2 Å dot spacing) seemed more

intelligible than the re-averaged map from Dot Model 5 (3 Å dot spacing). In the better map, the density at 2.6σ contour appeared to represent stacked beta-sheets and helices, and their connectivity. A model (since abandoned) was built by analysis of the connectivity of this map. In later tests of lower symmetry, it was observed that further small shifts of phases near the resolution limit would trigger re-interpretations of the density, such as re-inclining the cap helices. This led to re-examination of the averaging parameters.

The map used to construct the cpMVP model presented here resulted from two further parameter changes. After initial tests, the 48-fold multimer masks were tilted -13.68° from the orthogonal **Z** direction, but for part of the averaging history, the NCS 48-fold matrices for averaging represented a -13.5° tilt. The NCS matrices were corrected. Near the end of dot model refinement, the multimer averaging masks were drawn at the default 3 Å distance from the dots, and the solvent masks were made by assembly of the multimer masks. A new solvent mask was made with a 4 Å radius parameter, leaving the multimer masks 3 Å from the dot model boundaries. The previous best phases were used as starting values for a total of 800 cycles of DM, using these revised conditions. This phase set is part of PDB entry 2QZV. The map calculated from this phase set is shown in Fig. 3 and in panels of Fig 5.

Validation of the phasing processes. Throughout these low-resolution phasing trials and dot model refinements, the global statistics were poor indicators of progress. An initial R-factor can be deceptively high in the case of hyper-centric intensity statistics (estimated

maximum is 1 [11]). For randomized atoms in a centric space group, the R-factor would be 0.83 [12]. Although the vault density has become acentric, at the low resolution of this analysis, the geometrically patterned hollow vault results in almost hyper-centric intensity statistics. The solvent model is important in scaling together $\mathbf{F}_{observed}$ and $\mathbf{F}_{calculated}$ at low resolution [13]. MOLREP [8,14] calculated R-factors of more than 0.85 from its overlapped and mis-aligned (and over-weighted) models. The $\mathbf{F}_{calc}$ derived from inversion of the manually placed thin cryo-EM map (using SFALL [8]) gave an R-factor of 0.73 for reflections between 190-16Å (0.92 for 190-9Å). The R-factor from Dot Model 6 is 0.53 in the range of 200-9 Å ($\mathbf{F}_{calc}$ from SFALL, re-scaled with RSTATS [8]). Most statistics calculated by DM were optimistic even for unintelligible maps. DM reports correlation coefficients between NCS-related regions of each multimer-mask. For the density from the slow re-averaged Dot Model 6, these NCS correlations were about 0.8 for the 48-fold symmetric majority of the map, and about 0.9 for the 24-fold cap region. The electron-density map becomes less symmetric near lattice contacts (see Fig 3), as though the vault is not exactly round. The $\mathbf{F}_{calc}$ from DM incorporates contributions from the flat solvent model filling 82% of the cell, and resulted in an R-factor of 0.55 (after re-scaling with RSTATS). The correlation coefficient between $\mathbf{F}_{obs}$ and $\mathbf{F}_{calc}$ from DM is 0.76 in resolution range 190-9Å (from RSTATS). Intelligibility of the electron-density map indicated progress more than did the global statistics.

1. Isaacs NW, Agarwal RC (1985) Free atom insertion and refinement as a means of extending and refining phases. Methods Enzymol 115: 112-117.
2. Lamzin VS, Wilson KS (1993) Automated refinement of protein models. Acta Crystallogr D Biol Crystallogr 49: 129-147.

3. Perrakis A, Sixma TK, Wilson KS, Lamzin VS (1997) wARP: improvement and extension of crystallographic phases by weighted averaging of multiple-refined dummy atomic models. Acta Crystallogr D Biol Crystallogr 53: 448-455.
4. Kleywegt GJ, Jones TA (1996) xdlMAPMAN and xdlDATAMAN - Programs for reformatting, analysis and manipulation of biomacromolecular electron-density maps and reflection data sets. Acta CrystallogD 52: 826-828.
5. Kleywegt GJ, Jones TA (1999) Software for handling macromolecular envelopes. Acta CrystallogD 55: 941-944.
6. Kleywegt GJ, Jones TA (1994) Detection, delineation, measurement and display of cavities in macromolecular structures. Acta Crystallogr D Biol Crystallogr 50: 178-185.
7. McRee D (1999) Practical Protein Crystallography. San Diego: Academic Press.
8. CCP4 (1994) The CCP4 suite: programs for protein crystallography. Acta Crystallogr D Biol Crystallogr 50: 760-763.
9. Read RJ (1986) Improved Fourier coefficients for maps using phases from partial structures with errors. Acta Crystallog A 42: 140-149.
10. Cowtan K (1994) Joint CCP4 and ESF-EACBM Newsletter on Protein Crystallography 31: 34-38.
11. Douglas AS, Woolfson MM (1954) The maximum probable value of the reliability index for a hypercentric structure. Acta Crystallog 7: 517.
12. Wilson AJC (1950) Largest likely values for the reliability index. Acta Crystallog 3: 397-398.
13. Rees B, Jenner L, Yusupov M (2005) Bulk-solvent correction in large macromolecular structures. Acta Crystallogr D Biol Crystallogr 61: 1299-1301.
14. Vagin A, Teplyakov A (1997) MOLREP: an automated program for molecular replacement. JApplCryst 30: 1022-1025.
