# Supplementary material for: Draft Crystal Structure of the Vault Shell at 9-Å Resolution
Source: PLoS Biol. 2007 Nov 27;5(11):e318. doi: 10.1371/journal.pbio.0050318 (PMC2229873; doi:10.1371/journal.pbio.0050318)
Supplement: Text S9 — (92 KB PDF) [file pbio.0050318.sd010.pdf]

Text S9. Details of Model Building for Specific Domains

Domains 2 and 7, and assignment of enantiomer. Models were predicted for residues 26-87 and 357-404 using the ROSETTA algorithm [1-3]. The overall shapes of two of these models could be placed in the overall shapes in their corresponding electron densities (initially at 1.2σ contour) provided that the enantiomer of the phase set was flipped (phases were multiplied by -1). The vertical position of domain 7 was estimated from the number of dots in the dot models (Text S8). The volume of the dots was thought to be proportional to the number of amino acid residues (assuming that all 873 residues would be represented by density). In the initial partitioning shown in Fig. 1, domain 7 corresponds to density block 6. The initial models for domains 2 and 7 were later replaced with more credible models (Figs. 5b, 5e), but the shape-based enantiomer assignment was not changed.

Starting from the ROSETTA model for domain 2, the first two strands of the predicted 7-strand beta-sheet were flipped down to contact the bottom of the neighbor domain 2 to the right. The last strand of the domain 2 model was twisted and brought across the top of the model (with hydrophobic contacts) to join part of a second ROSETTA model. A 4-strand beta-sheet was predicted for residues 88-112. The first two strands, residues 88-101, were inserted into density of the same size unoccupied by the model up to residue 87, above the right neighbor domain 2. There was no electron density indication of how to connect the domain 2 model to the NMR-derived domain 3; residues 102-112 were excluded from the model.

Domain 7 (Fig. 5e) was adapted from a ROSETTA model primarily by interrupting the predicted β-sheet hydrogen bond pattern at pro 381. The upper domain 7 density and atoms reach down towards a change of chain direction due to pro 367 in the lower density.

Domain 1. The 96 copies of N-terminal domain 1 are located entirely inside the vault at its waist (Fig. 1). Models predicted for residues 4-54 contained beta-strands for residues 4-20, of the proper length to fit the N-terminal electron density. One model was chosen, and residues 4-25 excised from it, because residues 4 and 22 were already turned towards further density. N-terminal residues were manually appended to residue 4 approximately in beta-conformation. This extrapolation into un-occupied density placed cysteines 10T, 8T, 6T and 4T of the N-terminal tag adjacent to a local-only 2-fold symmetry axis (yellow bars in Fig. 5a), where they can disulfide bridge to Cys 10T, 8T, 4T and 6T of a non-equivalent MVP in the other vault half. The working model for the nested N-termini included cysteine side chains to verify plausibility of the disulfides, as shown in Fig. 5a.

NMR-derived domains 3, 4, and 5. The NMR substructure of domains 3 and 4 [4] was adapted to the vault electron density. The shape of the electron density approximately repeats in domains 3, 4, and 5 (Fig. 5c). The rat liver sequence for residues 113-221 was threaded onto the lowest-energy conformer of the NMR structure (PDB entry 1Y7X) using the SWISS-MODEL comparative modeling server [5]. The threaded model was cut between residues 166 and 167, and each domain was separately placed in density,

initially as shown in Fig. 5 of [4]. The models were rotated and translated to place their upper beta-sheets in the upper parts of the repeated density, with no left-right NCS collisions, and the termini pointed towards the domains above and below. Residues 222-276 of the rat sequence were threaded onto residues 167-221 of the threaded NMR model, and this module was placed in the third repeat density by analogy to the first two. These model placements left loops outside of density, while the lower density of each repeat contained not enough atoms. The bottom strands (residues 150-157, and 202-211) were flipped down and back into the bumps in the backgrounds of the lower densities. These density bumps were about the same size and shape as the flipped strands. The flexible loop of domain 3 (residues 126-139) was manually flipped and slightly re-shaped into the foreground of the lower density, forming hydrophobic contact to the same chain of MVP. The corresponding loop in domain 4 (residues 180-192) was flipped to the foreground lower density of the left neighbor MVP, forming hydrophobic contacts there. Partway through model-building, rat sequence 222-276 was re-threaded onto the modified domain 4 model. The threading algorithm could not fit residues 251-254 of domain 5 onto residues 198-200 of domain 4. The extruded residues 251-254 were manually rotated up into a foreground bump in the upper domain 5 density. This bump was not present in the other two repeat densities. The variable loop in domain 5 has since been flipped to contact the same MVP chain, as in domain 3. Domains 3, 4, and 5 were connected by manual bending of their termini (Fig. 5c). In retrospect, the underlying NMR model is suspicious (see *Validation* section, Text S1).

The ROSETTA algorithm operating on several sequence windows predicted a long beta hairpin for residues 280-305. Between domains 5 and 6, the electron density did not indicate where to place so many atoms. Residues 277-305 were excluded from the present MVP model, but would be in the vault interior if they could be placed in density.

Domain 6. The β-like foreground structure and first α-helix (Fig. 5d) originated in a ROSETTA prediction. The second helix was manually folded to match the length of the density. It contacts the first helix with hydrophobic surface, and exposes hydrophilic surface to solvent in the background.

Domains 8 and 9 were adapted from overlapping ROSETTA models. The predicted hydrogen bond patterns were manually disrupted at pro 420 (background helix in Fig. 5f; break in helix appears in higher contour density), and at prolines 445 and 448 terminating β-sheet patterns. These prolines are the nominal boundary between domains 8 and 9 (Table 1).

Domain 10. The ROSETTA-predicted segments for domain 10 were threaded through density (Fig. 5g). The third strand of the 3-strand bundle was manually placed to interleave hydrophobic side chains with the other two strands.

Domain 11. The helix of domain 11 (Fig. 5h) was manually templated onto ideal helix, starting from a fragmentary ROSETTA model. This helix (pro 565 to arg 597) almost exactly matches one of the predicted ranges for helix propensity, and forms favorable

interactions in all directions. Where the helix extends past domain 10 (after val 586), the bottom solvent-exposed surface becomes hydrophilic, while the top surface is hydrophobic, and contacts the bottom of domain 12.

Domain 12. The domain 12 starting model was a beta-sheet, but required some residue flips because of packing against its neighbors, and also required bending for prolines (Fig. 5i). Density for the lower two strands was better than for the top two strands, indicating increasing disorder in strands further from the shoulder structure. Lysine 621 and arg 623 in the middle strands are the dominant trypsin cleavage sites (data not shown).

Coiled-coil Domain 13, and crossover and cap disk structures of non-equivalent Domains 14a and 14b. The C-terminal parts of the cpMVP model were built by inspection of the electron density, initially as poly-alanine with arbitrary sequence numbering. These C-terminal models were iteratively revised to pack density, to minimize NCS collisions, and to maintain plausible backbone geometry, and only later discovered to be compatible with the MVP sequence (see *Validation*, Text S1). The electron density was initially pattern-less in the vertical part of the C-terminal cap region (density block 10 in Fig. 1, red domain 13 in Fig. 4). With dot model refinement, the surface of this density developed ridges and eventually tubes (at 2.6σ contour) that were used to guide placement of a stack of short poly-alanine helix models (model in Figs. 4 and 5j has sequence assigned).

Reasoning that similar structures would result from the identical sequences in the non-equivalent C-termini, the domain 14a and 14b models were built inside-out relative to each other. A poly-alanine "crossover" model was invented to reduce symmetry from 48-fold to 24-fold (model in Figs. 4 and 5k has sequence assigned). The MVP model is 48-fold symmetric from the N-termini at the waist to residue 715, near the top of the cap helices, 24-fold symmetric from 716 to 779 in the C-terminal cap disks. The electron-density evidence in the crossover zone consists of two rings between the 48-fold and 24-fold symmetric regions of the vault (bottom of Fig. 5k). The chain A and chain B models were built through the lower ring for a length of about 1.5 48-fold spacings in opposite directions. In the upper ring, both model chains return by about one 48-fold spacing to re-convene at the base of the 24-fold symmetric two-layer cap. At low resolution, NCS repetition of such a crossover structure would produce the two rings seen in the electron density.

The C-terminal cap disk portions of domains 14a and 14b (Figs. 4 and 5l) were constructed starting at their peripheries, where some texture was visible in the electron density. The electron density indicated that the MVP chains enter the C-terminal disks in opposite directions (evaluated at higher contour than shown at top of Fig. 5k). The inner C-terminal disk density (domain 14b) was more detailed than the outer disk density. The radius decreases as the MVP chains approach the C-termini of the modeled density. Poly-ala segments were inserted into the decreasing width per MVP as α-helix, 3_{10} helix, and beta-strand. The α-helical part of the inner disk model followed the inclination of the density. The rest of the inner C-terminal model was inserted, without benefit of detailed

density, to estimate how many more residues might fit. The outer C-terminal disk model (domain 14a) was built mostly by analogy to the inner disk; only the outermost helix could be inclined according to density. Beyond the C-termini of the present cpMVP model, the fragmentary electron density in both disks turns up, indicating the direction of the un-modeled remainder of the MVP chain (residues 780-861).

A high-resolution chain trace would likely require curved helices and beta-like strand to accommodate the decrease of radius on approach to the C-termini of the model. The cpMVP model leaves a hole at least 29 Å wide in each of its C-terminal disks. No residue can be appended in the same direction as the C-termini of the model because the 24 copies of the model in each disk are already too crowded with this thinnest of protein structures ((30π Å)/(24 MVP C-termini in each disk) = 3.9 Å per MVP C-terminus). A high-resolution model may require the C-termini to fray, before residue 779, to symmetry lower than 24-fold.

1. Bystroff C, Thorsson V, Baker D (2000) HMMSTR: a hidden Markov model for local sequence-structure correlations in proteins. J Mol Biol 301: 173-190.
2. Bystroff C, Shao Y (2002) Fully automated ab initio protein structure prediction using I-SITES, HMMSTR and ROSETTA. Bioinformatics 18 Suppl 1: S54-61.
3. Rohl CA, Strauss CE, Misura KM, Baker D (2004) Protein structure prediction using Rosetta. Methods Enzymol 383: 66-93.
4. Kozlov G, Vavelyuk O, Minailiuc O, Banville D, Gehring K, et al. (2006) Solution structure of a two-repeat fragment of major vault protein. J Mol Biol 356: 444-452.
5. Schwede T, Kopp J, Guex N, Peitsch MC (2003) SWISS-MODEL: an automated protein homology-modeling server. Nucleic Acids Res 31: 3381-3385.
