# Supplementary material for: Draft Crystal Structure of the Vault Shell at 9-Å Resolution
Source: PLoS Biol. 2007 Nov 27;5(11):e318. doi: 10.1371/journal.pbio.0050318 (PMC2229873; doi:10.1371/journal.pbio.0050318)
Supplement: Text S10 — (11 KB PDF) [file pbio.0050318.sd011.pdf]

Text S10. Details of energy minimization. Domains 1 and 2 (residues 3T to 101, Figs. 5a, 5b) were energy minimized with side chains packed against their left-right neighbors related by 48-fold NCS. The N-terminal cysteine tag sulfur atoms manually placed at the local 2-fold were held fixed (see Fig. 5a). After minimization, the side chains did not pack well up-down across this 2-fold between non-equivalent chains, and the manually-aligned hydrogen bonds at the 2-fold were not well preserved. The three NMR-derived domains (Fig. 5c, residues 113-276) were not covalently linked to the rest of the model, and were separately (due to manual interventions) energy minimized, using 48-fold NCS packing. Residues 306 to 715 of chain B were energy minimized using 48-fold NCS, with all atoms of ala 715 fixed. The C-termini of chains A and B were energy minimized, packed among 10 other copies of each chain related by 24-fold NCS (see Fig. 5l), and all copies of ala 715 were held fixed. The MVP dimer model (PDB entry 2QZV) was built by concatenation of the separate model files. Side chains in 2QZV were limited to beta-carbons because the low-resolution electron density did not support rotamer choices.
